# Clathrin adaptor AP-1–mediated Golgi export of amyloid precursor protein is crucial for the production of neurotoxic amyloid fragments

**DOI:** 10.1016/j.jbc.2022.102172

**Published:** 2022-06-23

**Authors:** Yunan C. Januário, Jessica Eden, Luan S. de Oliveira, Raffaella De Pace, Lucas A. Tavares, Mara E. da Silva-Januário, Vinícius B. Apolloni, Elise L. Wilby, Randolf Altmeyer, Patricia V. Burgos, Sonia A.L. Corrêa, David C. Gershlick, Luis L.P. daSilva

**Affiliations:** 1 Center for Virology Research, Ribeirão Preto Medical School, University of São Paulo, Ribeirão Preto, São Paulo, Brazil; 2 Department of Cell and Molecular Biology, Ribeirão Preto Medical School, University of São Paulo, Ribeirão Preto, São Paulo, Brazil; 3 Cambridge Institute for Medical Research, University of Cambridge, Cambridge, UK; 4 School of Pharmacy and Medical Sciences, University of Bradford, Bradford, UK; 5 Cell Biology and Neurobiology Branch, Eunice Kennedy Shriver National Institute of Child Health and Human Development, National Institutes of Health, Bethesda, Maryland, USA; 6 Statslab, Department of Pure Mathematics and Mathematical Statistics, University of Cambridgee, Cambridge, UK; 7 Centro de Biología Celular y Biomedicina (CEBICEM), Facultad de Medicina y Ciencia, Universidad San Sebastián, Santiago, Chile; 8 Center for Aging and Regeneration (CARE), Facultad de Ciencias Biológicas, Pontificia Universidad Católica de Chile, Santiago, Chile; 9 Department of Life Sciences, Faculty of Science and Engineering, Manchester Metropolitan University, Manchester, UK

**Keywords:** amyloid precursor protein, adaptor protein 1, protein trafficking (Golgi), endosome, Alzheimer’s disease, Aβ, amyloid-β, AD, Alzheimer’s disease, AICD, APP intracellular C-terminal domain, AP, adaptor protein, APP, amyloid precursor protein, BACE-1, beta-site APP cleaving enzyme 1, BFA, brefeldin A, Cas9, CRISPR-associated protein 9, CT, cytosolic tail, CTF, carboxyl-terminal fragment, DMEM, Dulbecco’s modified Eagle's medium, ER, endoplasmic reticulum, HA, hemagglutinin, HEK293, human embryonic kidney 293 cell line, HRP, horseradish peroxidase, PFA, paraformaldehyde, RCF, relative centrifugal force, RUSH, retention using selective hooks, RT, room temperature, SBP, streptavidin-binding peptide, SP, signal peptide, TGN, *trans*-Golgi network, Y2H, yeast two-hybrid

## Abstract

One of the hallmarks of Alzheimer’s disease is the accumulation of toxic amyloid-β (Aβ) peptides in extracellular plaques. The direct precursor of Aβ is the carboxyl-terminal fragment β (or C99) of the amyloid precursor protein (APP). C99 is detected at elevated levels in Alzheimer’s disease brains, and its intracellular accumulation has been linked to early neurotoxicity independently of Aβ. Despite this, the causes of increased C99 levels are poorly understood. Here, we demonstrate that APP interacts with the clathrin vesicle adaptor AP-1 (adaptor protein 1), and we map the interaction sites on both proteins. Using quantitative kinetic trafficking assays, established cell lines and primary neurons, we also show that this interaction is required for the transport of APP from the *trans*-Golgi network to endosomes. In addition, disrupting AP-1-mediated transport of APP alters APP processing and degradation, ultimately leading to increased C99 production and Aβ release. Our results indicate that AP-1 regulates the subcellular distribution of APP, altering its processing into neurotoxic fragments.

Alzheimer’s disease (AD) is characterized by the progressive loss of cognitive functions associated with learning and memory impairments. The AD brain typically presents an accumulation of intraneuronal fibrillary tangles and extracellular amyloid plaques (1, 2). These amyloid plaques are mainly composed of insoluble amyloid-β (Aβ) peptides, which are fragments of the amyloid precursor protein (APP) (3). APP may undergo non-amyloidogenic processing by α-secretases, generating a carboxyl-terminal fragment (CTF) known as CTFα or C83. Non-pathogenic fragments are subsequently generated upon γ-secretase cleavage. In contrast, Aβ production results from the amyloidogenic processing of APP by the β-secretase enzyme (BACE-1 [beta-site APP cleaving enzyme 1]). BACE-1 cleavage generates a longer CTF (CTFβ/C99) that is further processed by γ-secretase to produce Aβ peptides (4). The intracellular production/accumulation of C99 is, therefore, a prerequisite for Aβ biogenesis and extracellular amyloid plaque formation. A growing body of evidence has directly linked the intracellular accumulation of C99 to early neurotoxicity and cognitive dysfunction in AD onset (5, 6). A recent study indicates that intracellular accumulation of C99 in human neurons is the direct cause of lysosome function impairment (7) that is frequently observed in early onset AD (8).

APP and its processing secretases are transmembrane proteins synthesized in the endoplasmic reticulum (ER) that traffic through the secretory and endocytic pathways (6). The itinerary taken by these proteins within the endomembrane systems is crucial in determining the amyloidogenic processing of APP (6). The prolonged retention of APP or β-secretase at the Golgi apparatus or in early endosomes was shown to increase the pool of C99 and Aβ species (9, 10, 11, 12). Adaptor protein (AP) complexes are components of vesicle coats and critical for protein sorting in the late secretory pathway. There are five APs described in mammals, AP-1 to AP-5, and each adaptor selects a subset of proteins in specific compartments to be delivered to target membranes (13, 14). AP-1 is a clathrin adaptor complex that interacts with the cytoplasmic termini of membrane proteins and has been implicated in bidirectional protein trafficking between the *trans*-Golgi network (TGN) and early endosomes. AP-1 is also involved in polarized transport to the cell surface of neurons and epithelial cells (13). The selective transport of cargo mediated by AP-1 is crucial for cell homeostasis, as mutations in AP-1 subunits result in neurological diseases (15, 16).

The AP-1 complex is formed by four different subunits: two large (γ and β), one medium (μ1), and one small (σ1). The main function of this complex is to recruit transmembrane cargo and cytosolic proteins required for vesicle formation and transport (14). AP-1 is potentially the most diverse AP since three of its subunits present isoforms encoded by distinct genes. Specifically, there are two isoforms for the γ (γ1 and γ2), two for μ1 (μ1A and μ1B), and three for the σ1 (σ1A, σ1B, and σ1C) subunits (17). All these isoforms are ubiquitously present in different cell types, except for μ1B, which is specifically expressed in polarized epithelial cells (18). The μ1 subunit plays an essential role in cargo selection through a tyrosine-binding pocket that recognizes the cytosolic domains of transmembrane proteins containing a tyrosine-based YXXØ sorting signal (where X represents any amino acid and Ø a hydrophobic residue) (19). Interestingly, μ1B preferentially binds to a subset of noncanonical sorting signals in basolateral proteins, indicating that μ1A and μ1B isoforms may comprise functionally distinct AP-1 variants (19, 20). AP-1 complexes containing either μ1A or μ1B are frequently termed AP-1A and AP-1B, respectively.

The cytosolic tail (CT) of APP contains sorting motifs that mediate its interaction with the μ4 subunit of AP-4 (10) and a possible interaction with the AP-1 subunit isoforms μ1A and μ1B (21). It was shown that AP-1B, a variant that is not expressed in neurons, mediates the polarized trafficking of APP in epithelial cells (21). However, the functional role of the ubiquitously expressed AP-1A variant in APP trafficking and processing remains unknown. In this study, we developed a novel approach to monitor APP trafficking and cleavage in the secretory pathway, using a dual-tagged APP construct in a retention using selective hooks (RUSH) system that is amenable to flow cytometry, imaging, and protein biochemistry assays. Using these assays, we confirmed the importance of the APP CT on its distribution in the secretory pathway and have identified the amino acid residue Y682 as part of a sorting motif regulating APP anterograde transport and processing. We show that Y682 is crucial for APP interaction with μ1A, although it is not involved in AP-4 interaction (10). Functional analysis demonstrated that AP-1A is required for the efficient Golgi exit of APP and delivery to early endosomes. Consistently, the APP Y682A mutant that does not interact with AP-1A is retained in the TGN, and it is less efficiently transported to early endosomes. Disrupting APP–AP-1 interaction slows down the production of the Aβ precursor fragment C99, suggesting that APP processing by β-secretase in the Golgi is less efficient than in endosomes. Despite this, after extended periods, Golgi retention leads to intracellular accumulation of C99 and increased amyloid-β release. Taken together, our results demonstrate that AP-1 mediates the delivery of APP from the Golgi to early endosomes, directly affecting the processing and production of amyloid-β.

## Results

### The cytosolic tail of APP contains sorting motifs for anterograde transport in the secretory pathway

**Figure 1:**
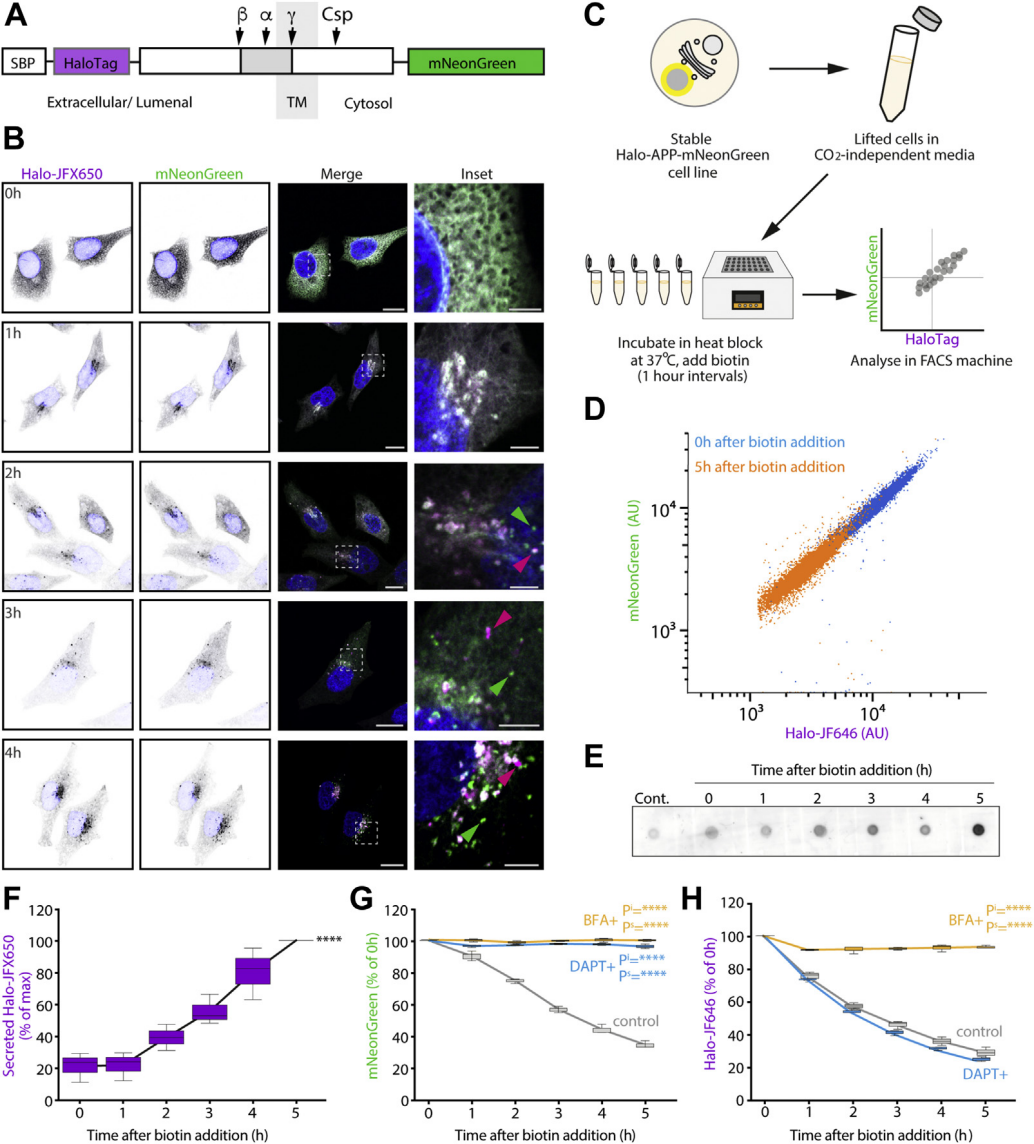
**Processing of Halo-APP-mNeonGreen can be quantitatively monitored using RUSH.***A*, schematic of dual-tagged APP RUSH construct with major APP cleavage sites for β-, α-, and γ-secretases and caspases (Csp) highlighted. *B*, confocal microscopy of Halo-APP-mNeonGreen over 4 h after biotin addition to induce ER export. *Blue* = nuclear DAPI staining. Images taken on a Zeiss 880 microscope at 63× magnification. *Main panels* scale bar represent 10 μm; *insets* scale bar represents 2.5 μm. *C*, schematic representation of RUSH APP workflow. Stable Halo-APP-mNeonGreen RUSH cells are lifted and incubated at 37 °C (after pre-treatment with appropriate drug). Biotin was added to cells for 0 to 5 h to induce ER export. After 5 h, fluorescent intensities were measured by flow cytometry as a readout of APP processing. *D*, mNeonGreen and Halo-JF646 fluorescence intensities at 0 and 5 h after ER export. *E*, immunodot blot of Halo-JFX650 fluorescence in culture medium over 5 h after Halo-APP-mNeonGreen is exported from the ER. *F*, quantification of Halo-JFX650 secretion after ER export of Halo-APP-mNeonGreen using a fluorescent plate reader assay. Halo-JFX650 fluorescence in the medium was quantified in a 96-well plate using a Clariostar plus plate reader. *G* and *H*, mNeonGreen (*G*) and Halo-JF646 (*H*) fluorescence intensities measured by FACS 0 to 5 h after ER export of Halo-APP-mNeonGreen, in cells pre-treated with 10 μg/ml BFA for 1 h or 25 μM DAPT (a γ-secretase inhibitor) for 24 h prior to inducing ER export. Fluorescence intensities are expressed as a percentage of the 0 h time point. ∗∗∗∗*p* ≤ 0.0001. Pᔆ values indicate significance of the slope. P^i^ values indicate significance of the Y-intercept. ∗∗∗∗*p* ≤ 0.0001; ∗∗*p* ≤ 0.01. APP, amyloid precursor protein; BFA, brefeldin A; DAPI, 4′,6-diamidino-2-phenylindole; ER, endoplasmic reticulum; ns, not significant; RUSH, retention using selective hooks.

To investigate the trafficking and cleavage of APP through the secretory pathway, we used the RUSH system (22). In this system, a reporter protein fused to a streptavidin-binding peptide (SBP) can be reversibly trapped in the ER by co-expression of streptavidin fused to an ER protein or ER retention signal. Upon the addition of biotin, the reporter-SBP chimaera is released from the ER hook and follows its itinerary within the secretory pathway in a synchronized fashion. To use this system to study APP trafficking, we generated a RUSH-competent APP reporter with dual fluorescent tags. On the lumenal N terminus is the SBP, to provide ER retention, alongside a HaloTag. On the cytosolic C terminus is an mNeonGreen tag (Fig. 1*A*). This construct is herein referred to as Halo-APP-mNeonGreen. The presence of the two fluorescent proteins at opposing ends of APP allows monitoring of APP cleavage in live cells. The cells used for this experiment are HeLa, which have the machinery to process APP (23) and produce the CTFs (10). Through the addition of soluble biotin, Halo-APP-mNeonGreen is exported from the ER, and its trafficking and processing can be monitored.

HeLa cells stably expressing streptavidin-KDEL and Halo-APP-mNeonGreen were stained with fluorescent Halo-JFX650 ligand, treated with biotin and imaged every hour for 4 h. Before the addition of biotin, both Halo-JFX650 and mNeonGreen were co-localized in a reticular pattern at the ER (Fig. 1*B*). After biotin addition, both Halo-JFX650 and mNeonGreen accumulated in the juxtanuclear region over time. As APP was synchronously trafficked through the cell, independent Halo-JFX650 and mNeonGreen puncta became visible. This indicates that APP cleavage had occurred, and the C-terminal and N-terminal APP fragments had been independently sorted into different compartments in the cell.

As APP is trafficked through the cell, it is cleaved into different fragments depending on its co-localization with various secretases in different subcellular environments. Luminal APP fragments are lost to the extracellular space (24, 25), whilst the cytosolic fragments are degraded intracellularly (26). We, therefore, reasoned that the loss of both fluorescent tags could be monitored by flow cytometry using the RUSH system. HeLa cells stably expressing streptavidin-KDEL and Halo-APP-mNeonGreen were incubated in suspension at 37 °C (Fig. 1*C*). The release of Halo-APP-mNeonGreen from the ER was induced through the addition of biotin to the cell culture media, and its trafficking was allowed to proceed for 0 to 5 h. The Halo-JF646 and mNeonGreen fluorescence of each cell was then measured by flow cytometry (Fig. 1*D*). As expected, with biotin addition, there is a time-dependent decrease in both mNeonGreen-tagged CTFs and Halo-tagged N-terminal fragments. The kinetics of APP trafficking observed here match those of other systems (11).

We hypothesized that the observed decrease in HaloTag fluorescence was due to secretion of the luminal APP fragment. To test this, cells expressing Halo-APP-mNeonGreen were seeded in a 6-well plate, and ER release was induced in each well for 0 to 5 h. The medium was then removed, concentrated, and the Halo-JFX650 fluorescence was visualized on a nitrocellulose membrane to assess levels of Halo-JFX650 secretion during APP trafficking and processing (Fig. 1*E*). Halo-JFX650 secretion was also quantified using a fluorescent plate reader assay (Fig. 1*F*). Using both assays, we observed a steady increase in Halo-JFX650 fluorescence in the conditioned medium over 5 h after ER export. This indicates that the Halo-tagged N terminus of APP is cleaved and secreted to the extracellular space.

Together with the secretion of the Halo-tagged N terminus, the amyloidogenic and non-amyloidogenic proteolytic pathway generates the C-terminal membrane fragments C99 and C83, respectively. γ-secretase enzyme liberates from these fragments a soluble APP intracellular C-terminal domain (AICD) in the cytosol. C99, C83, and AICD have been shown to undergo intracellular degradation by several different pathways (26, 27, 28). To test whether these fragments are degraded by the proteasome, cells were pre-treated with MG132, a proteasome inhibitor, before the release of Halo-APP-mNeonGreen from the ER (Fig. S1*A*). Proteasome inhibition by MG132 prevented the loss of C-terminal mNeonGreen fluorescence, indicating that these APP fragments generated in our system are indeed degraded by the proteasome. As expected, MG132 treatment did not affect the N-terminal HaloTag-APP fluorescence. In summary, the time-dependent decrease observed in mNeonGreen fluorescence is, in part, due to proteasomal degradation of the CTFs generated in amyloidogenic and non-amyloidogenic processing, whilst the decrease observed in HaloTag fluorescence during this time is due to secretion of the N-terminal fragment into the extracellular space.

To demonstrate that this assay physiologically recapitulated endogenous APP trafficking, we used the RUSH system in combination with several molecular inhibitors. To determine whether γ-secretase is involved in the processing of the cytosolic mNeonGreen fragment, cells were pre-treated with DAPT (Sigma–Aldrich), a γ-secretase inhibitor, before ER release. This caused stabilization of the CTF-mNeonGreen fluorescence, whilst the reduction in Halo-JF646 fluorescence was unaffected (Fig. 1, *G* and *H*). This is in agreement with previous evidence demonstrating that DAPT treatment precludes AICD formation and subsequent processing and degradation. Importantly, this demonstrates that the loss of mNeonGreen observed using this assay is a direct effect of APP processing.

To determine if APP processing took place after Golgi exit, we pre-treated cells with brefeldin A (BFA) for 1 h before release from the ER (Fig. 1, *G* and *H*). BFA inhibits the GTPase exchange factor resulting in Golgi tubulation and the redistribution of Golgi-localized and secretory proteins into the ER (29, 30). In the presence of BFA, mNeonGreen and Halo-JF646 fluorescence were stabilized compared with control samples. To confirm this using an orthogonal approach, we incubated cells at 20 °C to prevent protein export from the Golgi (31) (Fig. S1*B*). Blocking Golgi export by both methods prevented the decrease in mNeonGreen and Halo-JF646 fluorescence, indicating that efficient APP processing takes place after its exit from the Golgi.

In summary, the dual-tagged Halo-APP-mNeonGreen RUSH system recapitulates both the kinetics, trafficking pathways, and pharmacological dependence of endogenous APP and represents a novel assay to monitor APP trafficking and processing.

### The Y682 on the APP CT prevents Golgi accumulation of APP

**Figure 2:**
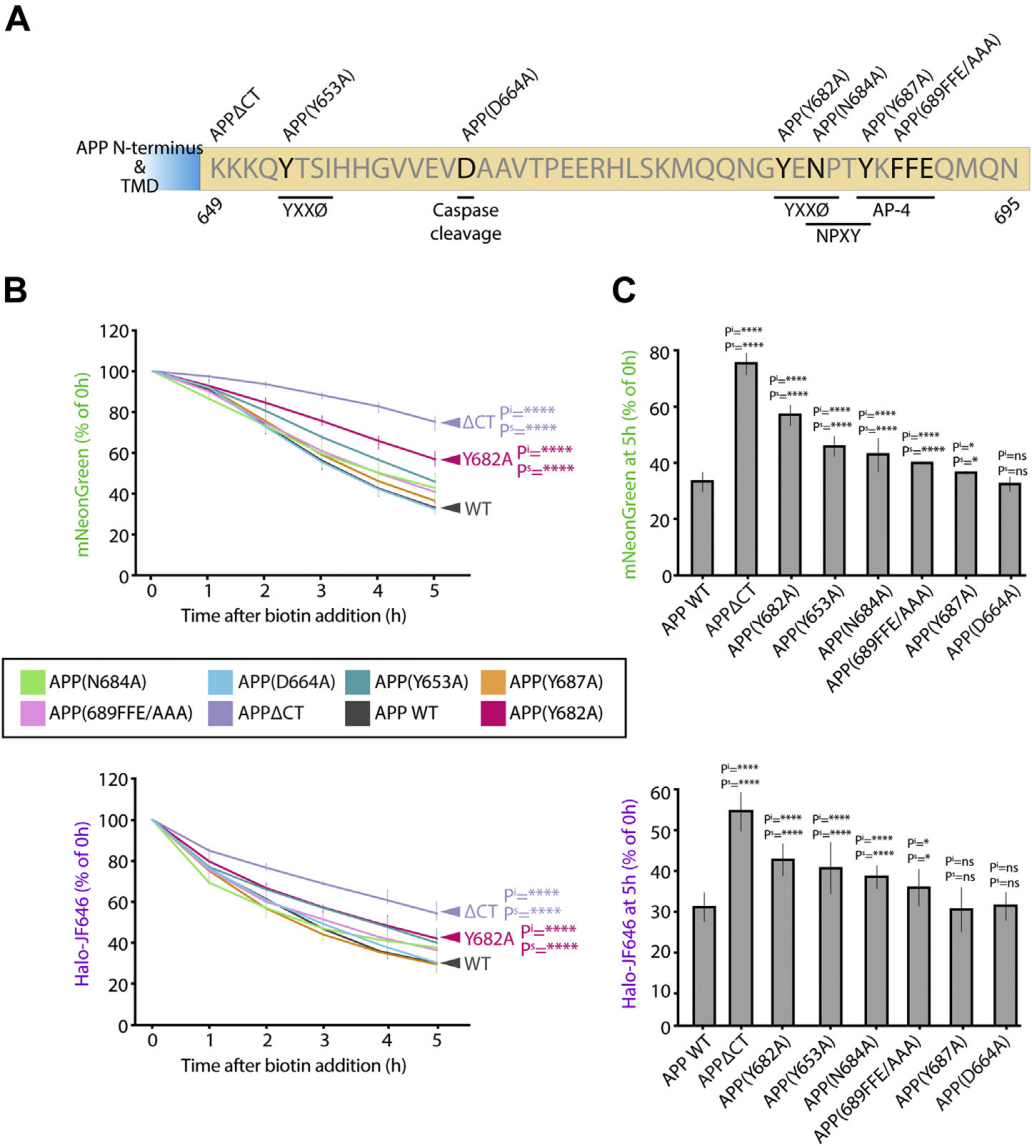
**The APP cytosolic tail (CT) contains sorting motifs that are important for its trafficking and processing.***A*, schematic of point mutations introduced in the CT of APP (649–695). Residues are numbered according to APP isoform 695. Important consensus motifs and binding sites have been highlighted. *B*, mNeonGreen and Halo-JF646 fluorescence levels of the APP CT mutants measured for 5 h after ER export. Fluorescence intensities are expressed as a percentage of the 0 h time point. *C*, mNeonGreen and Halo-JF646 fluorescence levels 5 h after ER export. Pᔆ values indicate significance of the slope. P^i^ values indicate significance of the Y-intercept. ∗∗∗∗*p* ≤ 0.0001; ∗∗*p* ≤ 0.01. APP, amyloid precursor protein; ns, not significant.

Previous literature indicates that the short CT of APP is important for its interactions with adaptor complexes through the presence of several tyrosine-based sorting motifs such as YXXØ, a well-characterized AP-1/2 recognition site (32), and YKFEE, an AP-4 binding site (10). To elucidate functionally important APP interactors, we first identified potential adaptor-binding sites in the CT of APP (Fig. 2*A*). Using an unbiased approach, we generated seven constructs with specific mutations in the CT, each designed to perturb a potentially important trafficking motif. A stable clonal cell line was generated for each mutant, and its processing was monitored after ER release using the RUSH system, as described previously. In the positive control, a mutant missing the whole CT, we see a significant decrease in the loss of both mNeonGreen and Halo-JF646, indicating a defect in trafficking and subsequent processing. The mutation at the caspase cleavage site (D664A) (33) had no detectable effect on either trafficking or processing and phenocopies the WT APP tail in this system. We see a significant decrease in the loss of both mNeonGreen and Halo-JF646 with different degrees of severity for most of the other trafficking motif mutations, aside from Y687A, which was not significant for the loss of Halo-JF646. Aside from the positive control (APPΔCT), APPY 682A had the most severe effect on APP processing (Fig. 2, *B* and *C*), indicating that this YXXØ motif is essential for the proper anterograde trafficking and processing of APP.

**Figure 3:**
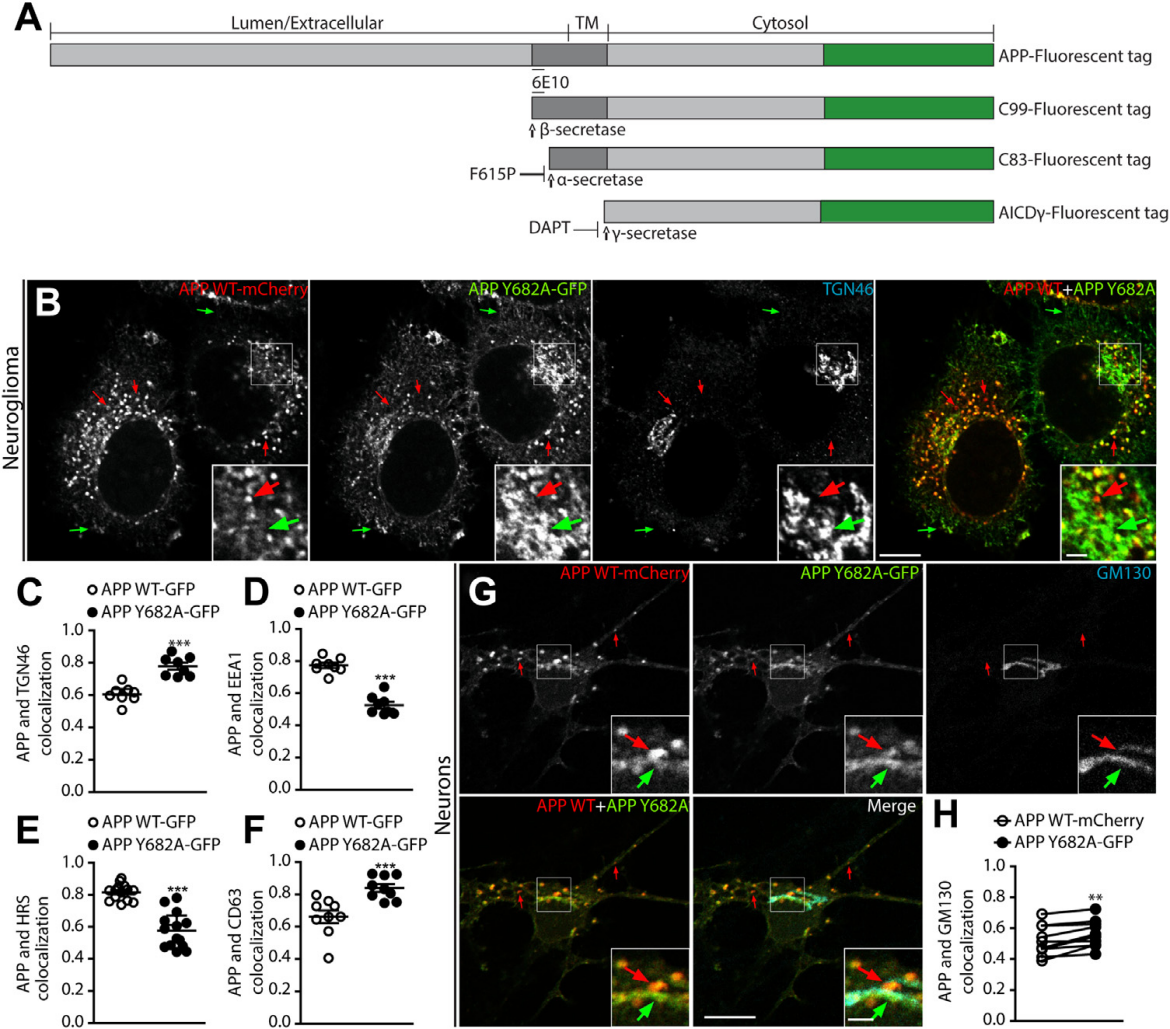
**Residue Y682 of the APP tail is essential for proper localization of APP.***A*, schematic representation of APP fused with either GFP or mCherry at the C terminus and its processing products. APP fragments after processing include C99, C83, and AICD-γ, all fused with GFP or mCherry. The recognition site of 6E10 antibody is indicated in full-length APP and C99. APP was expressed with a F615P substitution to reduce α-secretase cleavage. DAPT was used to inhibit γ-secretase activity and facilitate C99 fragment visualization. *B*, confocal microscopy images of H4 neuroglioma cells co-transfected with APP WT-mCherry and APP Y682A-GFP and immunolabeled with anti-TGN46. *Green arrows* indicate puncta exclusively of APP/CTFs Y682A-GFP. *Red arrows* highlight puncta with only APP/CTFs WT-mCherry. *C*–*F*, APP/CTFs WT-GFP and APP/CTFs Y682A-GFP co-localization with markers measured using Pearson’s coefficient. Values represent mean ± SEM from at least eight different cells. *G*, rat cortical neurons co-transfected with APP WT-mCherry and APP Y682A-GFP and immunolabeled with anti-GM130. *H*, APP/CTFs WT-mCherry and APP/CTFs Y682A-GFP co-localization compared in the same transfected neuron using Pearson’s coefficient. Merge channel is the combination of APP WT-mCherry, APP Y682A-GFP, and GM130 channels. *Main panels* scale bar represents 10 μm; *insets* (2×) scale bar represents 2.5 μm. ∗∗*p* ≤ 0.01; ∗∗∗*p* ≤ 0.001. Statistical significance was calculated by two-tailed Student's *t* test in *C*–*F* and *H*. APP, amyloid precursor protein; TGN, *trans*-Golgi network.

To further characterize the requirement of Y682 in APP trafficking, we co-expressed an APP WT-mCherry and an APP Y682A-GFP construct (Fig. 3*A*) in H4 neuroglioma cells and directly compared their distribution pattern in the same cell. We observed that APP/CTFs Y682A is markedly present at the juxtanuclear region and the cell surface, differently from the distribution of WT APP that was mostly found in cytosolic punctate structures (Fig. 3*B*). Furthermore, we characterized the distribution of APP/CTFs Y682A and APP/CTFs WT for different endogenous markers of the late secretory pathway, such as TGN46 (TGN), EEA1 and HRS (early endosomes), and CD63 (late endosomes). We observed that APP/CTFs Y682A accumulated at the juxtanuclear region and presented a significantly increased co-localization with the TGN46 marker (Figs. S2*A* and 3*C*). In addition, we found reduced amounts of APP/CTFs Y682A signal associated with the early endosomal proteins EEA1 and HRS, compared with WT APP/CTFs (Figs. S2, *B* and *C* and 3, *D*–*E*). Finally, we observed that APP/CTFs Y682A showed a significant increase in its association with the endolysosomal protein CD63 in comparison to WT APP/CTFs (Figs. S2*D* and 3*F*). Last, we confirmed the increased co-localization of APP/CTFs Y682A with the Golgi protein GM130 at the juxtanuclear region of primary neurons (Fig. 3, *G* and *H*), in comparison to WT APP/CTFs-mCherry. Together, these results indicate that the Y682 residue on APP is part of a sorting signal for reduced Golgi/TGN residency of APP/CTFs.

### APP interacts with both μ1A and μ1B subunits of AP-1 *via* multiple contact points

**Figure 4:**
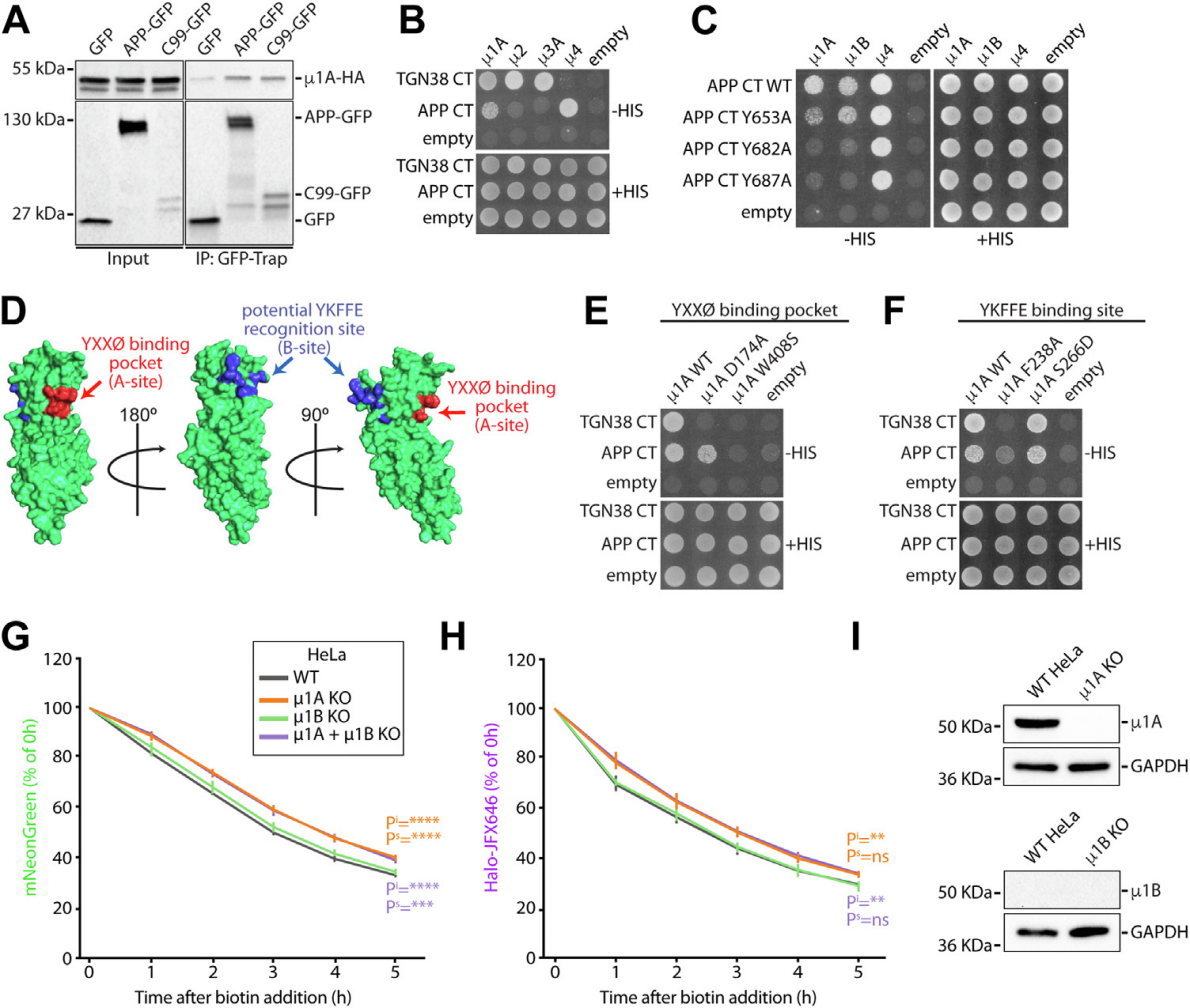
**Mapping of μ1A and APP tail residues involved in AP-1–APP interaction.***A*, GFP-Trap immunoprecipitation from cells co-expressing either GFP, C99-GFP, or APP-GFP with μ1A-HA. *B*, Y2H assay of APP cytosolic tail with the medium subunit from AP-1 to AP-4. Yeast growth in medium without histidine (−HIS) indicates that there is an interaction between proteins. TGN38 tail was used as a positive control for interactions with μ1A, μ2, and μ3. Empty represents the plasmid required for yeast transformation but with no protein expressed. *C*, Y2H assay between APP tail with tyrosine point mutations and subunits μ1A, μ1B, and μ4. *D*, μ1A C terminus 3D structure (Protein Data Bank ID: 1W63). *Red* indicates tyrosine-binding pocket (YXXØ), *blue* indicates potential APP YKFFE recognition sequence (a homologous μ4-binding site), modified from Ref. (36). *E*, Y2H of APP tail interaction with μ1A containing two-point mutations in the tyrosine-binding sites (D174A and W408S). *F*, Y2H of APP tail interaction with μ1A containing point mutations in the homologous μ4-binding site (μ1A F238A and μ1A S266D). *G* and *H*, transient CRISPR KOs of μ1A, μ1B, and μ1A + μ1B in Halo-APP-mNeonGreen RUSH cells. mNeonGreen and Halo-JFX646 fluorescence levels measured every hour for 5 h after APP ER export using flow cytometry. Fluorescence intensities expressed as a percentage of the 0 h time point. ∗∗∗∗*p* ≤ 0.0001; ∗∗∗*p* ≤ 0.001; ∗∗*p* ≤ 0.01. Pᔆ values indicate significance of the slope. P^i^ values indicate significance of the Y-intercept. *I*, Western blots to assess efficiency of the μ1A and μ1B CRISPR knockouts. Control is WT HeLa cells (where μ1B is not expressed). GAPDH was used as a loading control. AP-1, adaptor protein; APP, amyloid precursor protein; ER, endoplasmic reticulum; HA, hemagglutinin; ns, not significant; RUSH, retention using selective hooks; TGN, *trans*-Golgi network; Y2H, yeast two-hybrid.

The results presented previously indicate that the APP CT contains information for efficient anterograde transport and that Y682A is crucial in this process. AP-1A is known to mediate transport between the TGN and endosomes (13), and its interaction with APP has been previously shown (21). However, the functional relevance of this interaction has not been elucidated. Initially, we sought to confirm the APP–μ1A interaction using co-immunoprecipitation assays. We co-expressed hemagglutinin (HA)-tagged μ1A together with either GFP, APP-GFP, or C99-GFP constructs (Fig. 3*A*) in human embryonic kidney 293 (HEK293) cells (for a high yield of exogenous protein production) and used GFP-trap beads to pulldown GFP from cell extracts. μ1A co-immunoprecipitated with APP-GFP and C99-GFP but not with GFP alone (Fig. 4*A*).

As the Y682 mutant displayed the strongest defect in anterograde transport (Fig. 2*B*), we sought to test whether this residue is involved in AP-1 interaction. We used yeast two-hybrid (Y2H) interaction assays to identify the sequence requirements for the APP–μ1A interaction (Fig. S3*A*). In these experiments, we used the CT of TGN38, a prototypical μ1A interactor (34), as a positive control. Initially, we confirmed the interaction of the APP CT with μ1A as well as the previously reported interaction with μ4 (10) (Fig. 4*B*). In contrast, the APP CT did not interact with μ2 (AP-2) or μ3 (AP-3), subunits that showed a strong interaction with the TGN38 CT (Fig. 4*B*). We then analyzed the role of each tyrosine residue within the APP CT in its interaction with μ1A and μ1B. Y2H assays showed that APP Y653A substitution partially reduces the affinity to μ1A and μ1B (Fig. 4*C*). Similarly, a minor reduction in the interaction with μ1A and μ1B was observed with the APP I656A mutant and with APP bearing double Y653A/I656A mutations (Fig. S3*B*), indicating that the canonical _653_YXXØ_656_ motif in the APP CT is not essential for interaction with μ1A or μ1B. In contrast, we observed that the Y682A and Y687A substitutions abrogate the interaction with both μ1A and μ1B (Fig. 4*C*). These substitutions did not prevent the interaction with μ4 (Fig. 4*C*), confirming previous observations (10), although Y687 was proposed to be marginally involved with μ4 interaction (10). Interestingly, the F690 residue within the _687_YKFFE_692_ motif, necessary for μ4 interaction (10), is also required for APP interaction with μ1A (Fig. S3, *C* and *D*). Together, these results indicate diverse and conserved sequence requirements for APP interactions with μ1A or μ4. In addition, it reveals that the APP Y682A is a useful tool to study the function of AP-1 interaction while preserving the interaction with AP-4.

Our findings showed that different motifs present in the APP CT are required for the interaction with μ1A. We, therefore, sought to identify the APP-binding site in μ1A. The C-terminal domain of μ subunits is known to contain two distinct interaction sites for tyrosine-based sorting motifs located on opposite surfaces of the molecule (35, 36, 37) (Fig. 4*D*). The so-called A-site, also known as the tyrosine-binding pocket, typically recognizes the canonical YXXØ motifs, whereas the B-site was originally discovered in μ4 as the APP YKFFE-recognition site (10, 38). To test the importance of the μ1A A-site in APP interaction, we performed Y2H assays using μ1A carrying point mutations previously reported to abolish the interaction with YXXØ signals, specifically D174A or W408S (32, 39). As expected, the interaction of the TGN38 CT with μ1A was lost with mutations in both residues (Fig. 4*E*) (40). Interestingly, we found that the interaction of APP with μ1A was abolished by the W408S substitution (Fig. 4*E*), suggesting that the A-site in μ1A is required for this interaction. Based on the structural homology to μ4, we also analyzed the importance of a putative B-site in μ1A, using μ1A mutants carrying substitutions in F238A or S266D residues (37). The μ1A F238A substitution reduced the interaction with the APP CT, whilst the S266D substitution had no effect (Fig. 4*F*). Similarly, the TGN38 CT interaction with μ1A requires both the A-site (Fig. 4*E*) (40) and the B-site (Fig. 4*F*). These results demonstrate that the B-site in μ1A is required for the interaction with APP and TGN38.

To investigate the role of AP-1 in anterograde trafficking of APP, we generated CRISPR/CRISPR-associated protein 9 (Cas9) KOs for both AP1μ1A and μ1B in our stable Halo-APP-mNeonGreen HeLa RUSH system. Using the fluorescence-activated cell sorting–based approach described previously (Fig. 1*C*), we observed a significant decrease in the loss of both mNeonGreen and Halo-JF646 fluorescence in μ1A KO cells (Fig. 4, *G*–*I*), a behavior that was similar to the APP Y682A mutation in parental WT cells. We validated the AP1μ1A KO in this HeLa cell line by Western blotting (Fig. 4*I*); however, we were unable to detect μ1B by Western blotting in the control WT HeLa cell line, supporting previous data that indicate μ1B is not expressed in HeLa cells (18).

### Depletion of AP-1 increases the localization of APP in the TGN

**Figure 5:**
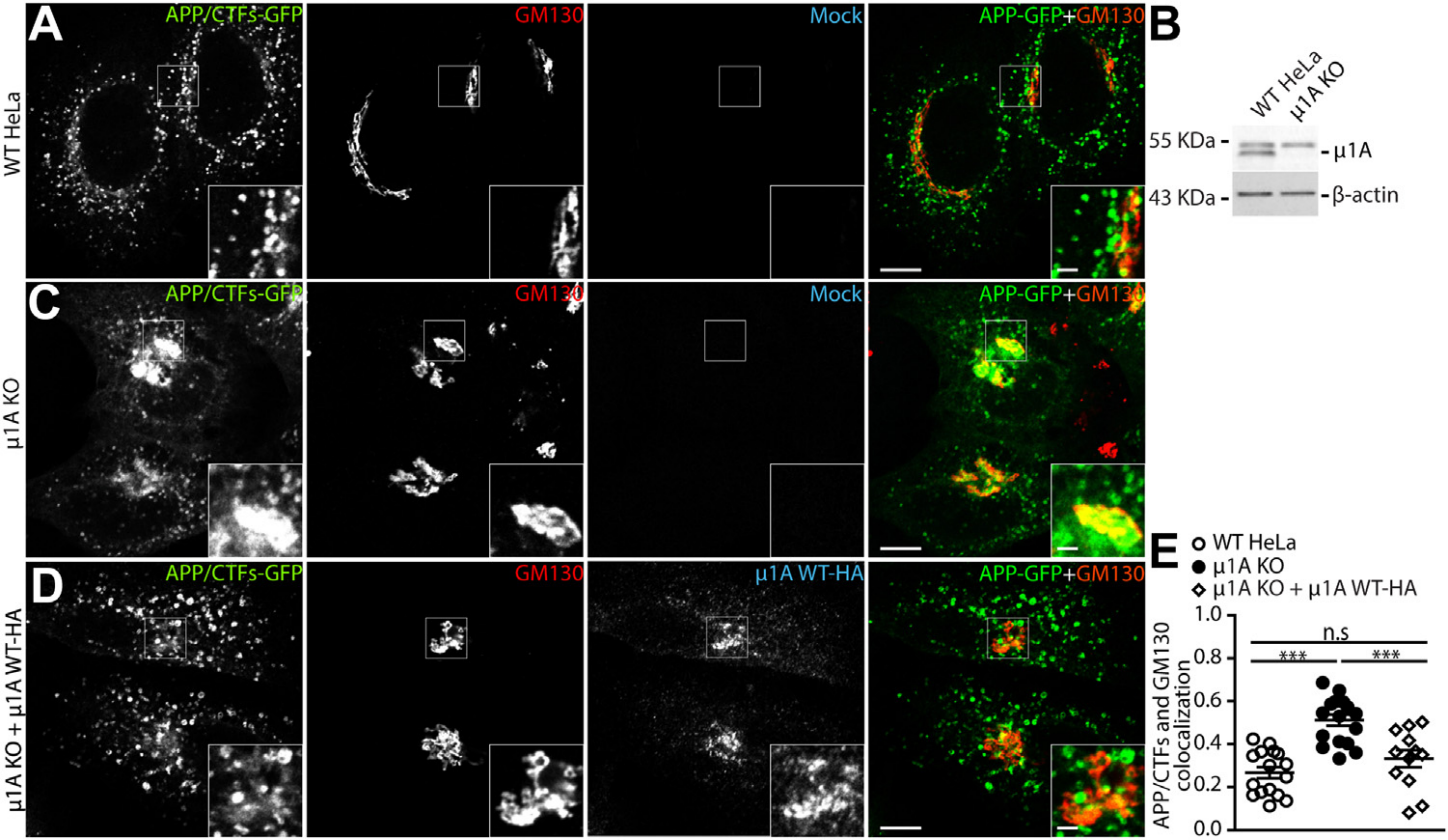
**The absence of μ1A subunit increases APP association with the Golgi.***A*, confocal microscopy images of HeLa cells transiently expressing APP-GFP, immunolabeled to anti-GM130 and anti-HA. *B*, Western blot to confirm KO of μ1A in HeLa cells. β-actin was used as a loading control. *C*, μ1A KO HeLa cells transiently expressing APP-GFP and immunolabeled to anti-GM130 and anti-HA. *D*, μ1A KO HeLa cells transfected with APP-GFP and μ1A WT-HA to rescue AP-1 function, immunolabeled with anti-GM130 and anti-HA. *E*, APP-GFP/CTFs and GM130 co-localization measured using Fiji software with Pearson’s coefficient. Values represent mean ± SEM from at least 11 different cells. The merge channel is a combination of APP-GFP and GM130 channels. *Main panels* scale bar represents 10 μm; *insets* (2×) scale bar represents 2.5 μm. ∗∗∗*p* ≤ 0.001. Statistical significance was calculated by one-way ANOVA followed by Tukey post-test. APP, amyloid precursor protein; CTF, carboxyl-terminal fragment; HA, hemagglutinin; ns, not significant.

The requirement of AP-1 in APP trafficking was confirmed in HeLa cells expressing APP-GFP (Fig. 3*A*). As expected, APP/CTFs-GFP was mostly present in dispersed punctate structures in control HeLa cells (Fig. 5*A*). In contrast, APP/CTFs-GFP was accumulated in the perinuclear region in μ1A CRISPR/Cas9 KO HeLa cells (20), where it co-localizes with the Golgi protein GM130 (Fig. 5, *B* and *C*). This change in localization was rescued in μ1A KO cells expressing exogenous HA-tagged μ1A (Fig. 5, *D* and *E*).

**Figure 6:**
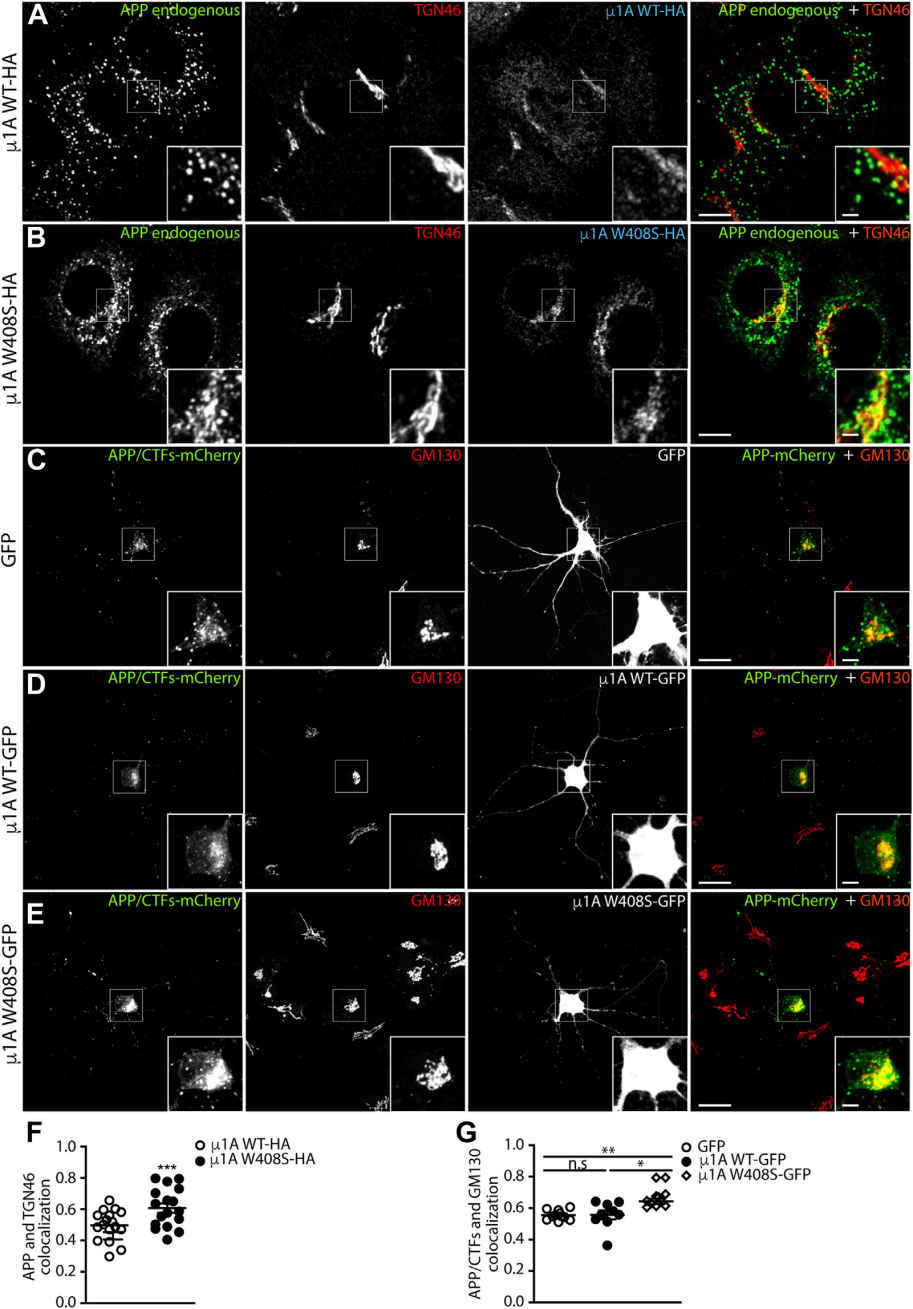
**Expression of dominant-negative μ1A W408S increases APP/CTF association with Golgi markers in H4 cells and neurons.***A* and *B*, confocal microscopy images of H4 cells transfected with μ1A WT-HA or μ1A W408S-HA, immunolabeled with anti-C99 (to stain endogenous APP and C99), anti-HA, and anti-TGN46 antibodies. Merge channel is a combination of APP/C99 and TGN46 channels. *C*–*E*, rat cortical neurons transfected with APP-mCherry in combination with GFP (*C*), μ1A WT-GFP (*D*), or μ1A W408S-GFP (*E*). Cells were immunolabeled with anti-GM130. *F*, endogenous APP/C99 and TGN46 co-localization in H4 cells expressing μ1A WT-HA or μ1A W408S-HA was measured using Fiji software with Pearson’s coefficient. Values represent mean ± SEM from at least 15 different cells. *G*, APP/CTFs-mCherry and GM130 co-localization in neurons expressing μ1A W408S-GFP, μ1A WT-GFP, or GFP alone. Values represent mean ± SEM from at least eight different cells. *Main panels* scale bar represent 10 μm; *insets* (2×) scale bar represents 2.5 μm. ∗*p* ≤ 0.05; ∗∗*p* ≤ 0.01; and ∗∗∗*p* ≤ 0.001. Statistical significance was calculated by two-tailed Student’s *t* test in *F* and one-way ANOVA followed by Tukey post-test in *G*. APP, amyloid precursor protein; CTF, carboxyl-terminal fragment; HA, hemagglutinin; TGN, *trans*-Golgi network.

To confirm the function of AP-1 in APP trafficking in different systems, we first used RNAi to knockdown the γ1 subunit of AP-1 in H4 human neuroglioma cells and analyzed the subcellular distribution of endogenous APP. We observed that whilst in control conditions, APP is mostly present in punctate structures dispersed in the cytosol (Fig. S4*A*), knockdown of AP-1 redistributes APP to the juxtanuclear region, where it co-localizes with the TGN marker TGN46 (Fig. S4, *B*–*D*). As an alternative approach to testing the importance of functional AP-1 in APP trafficking, we over-expressed the μ1A W408S mutant in H4 cells. This mutant is efficiently incorporated into the AP-1 complex (40) and acts as a dominant negative of μ1A-dependent AP-1 cargo transport, preventing the interaction with APP (20, 40). In comparison to μ1A WT, over-expression of μ1A W408S increased the endogenous APP signal (detected with an anti-C99 antibody) in the juxtanuclear area in close association with TGN46 (Fig. 6, *A*, *B* and *F*). Finally, to test whether the interaction of APP with μ1A is functionally relevant in neurons, we co-expressed APP-mCherry with GFP, μ1A WT-GFP, or μ1A W408S-GFP in primary rat cortical neurons at 12 days *in vitro*. APP/CTFs-mCherry was mostly localized in punctate structures at the cell body in either GFP or μ1A WT-GFP–expressing neurons (Fig. 6, *C*, *D* and *G*). In neurons co-expressing μ1A W408S-GFP, APP/CTFs-mCherry appeared more concentrated in the juxtanuclear area and showed increased co-localization with the Golgi marker GM130 (Fig. 6, *E* and *G*).

Together, the results establish that AP-1 acts as a piece of essential sorting machinery in controlling the distribution of APP within the secretory pathway.

### AP-1 mediates the efficient exit of APP from the Golgi and arrival at the early endosomes

To test if AP-1 mediates anterograde transport of APP from the Golgi to the early endosomes, we monitored APP trafficking in the secretory pathway using the RUSH system shown in Figure 1. In this case, APP is fused to an SBP and mCherry at the N terminus and GFP at the C terminus (therein termed mCherry-APP-GFP; Fig. S5*A*). We transfected WT HeLa and μ1A KO cells, stably expressing streptavidin-KDEL hook, with the RUSH mCherry-APP-GFP construct and followed both mCherry and GFP fluorescence before and after biotin addition. Before biotin treatment, mCherry/GFP fluorescence was found to co-localize in a reticular pattern in both WT and KO cells (Fig. S5, *B* and *G*, 0 min). Upon biotin addition, mCherry/GFP begin to accumulate in the juxtanuclear region in both cell populations (Fig. S5, *C*, *D*, *H* and *I*, 15–30 min). Intense juxtanuclear localization was followed by the display of mCherry/GFP punctate structures, which were more evident in WT cells compared with μ1A KO cells (Fig. S5, 60–120 min). Interestingly, μ1A KO cells presented a higher number of discrete puncta containing mCherry alone compared with WT cells (Fig. S5, *E*, *F*, *J* and *K*, *red arrows*), suggesting that in μ1A KO cells, N-terminal fragments may leave the Golgi more efficiently than CTFs. In addition, this observation strongly indicates that the pool of the juxtanuclear accumulation could correspond to CTFs rather than full-length APP.

**Figure 7:**
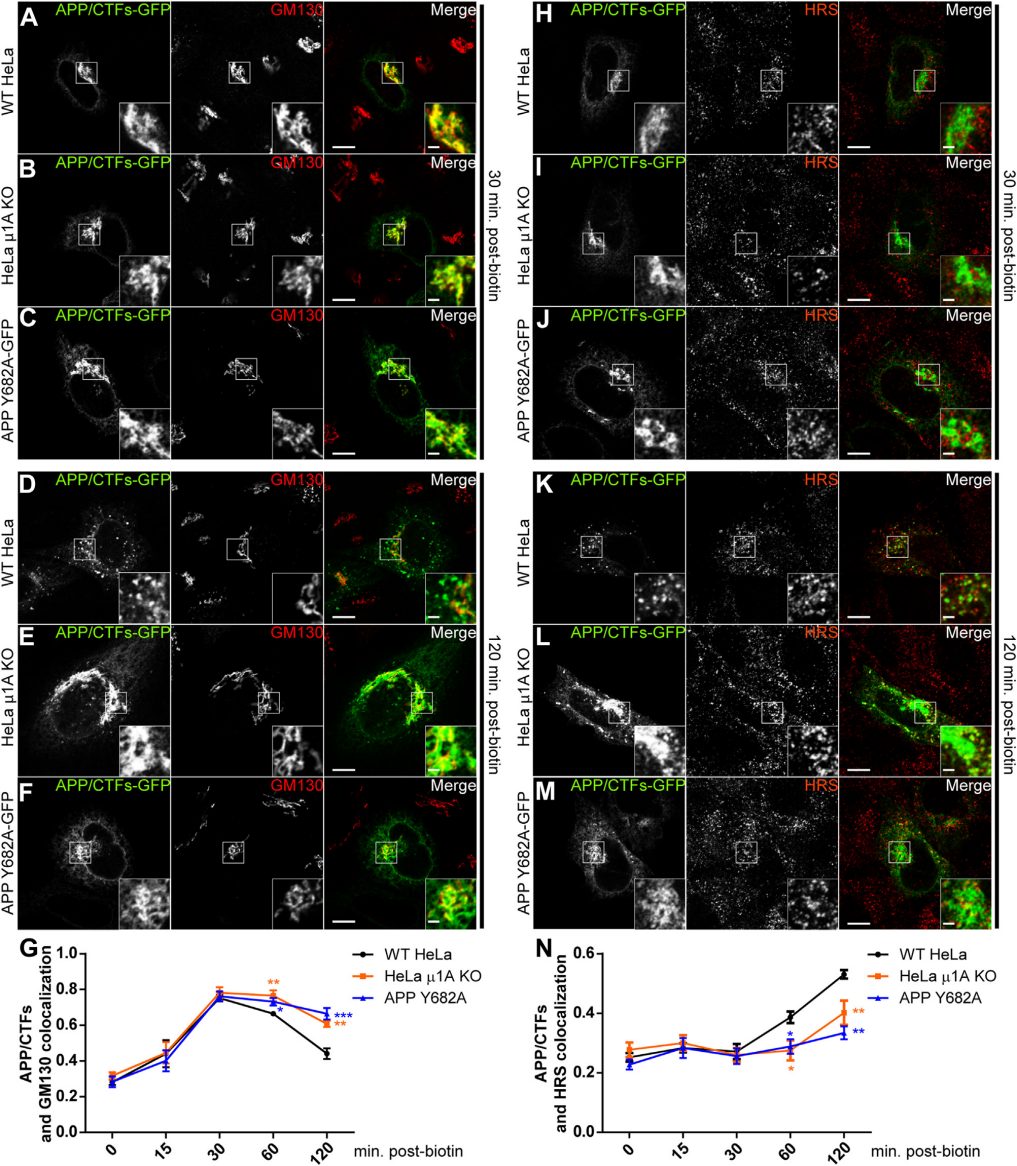
**μ1A KO delays APP export from the Golgi and its anterograde transport to early endosomes.** Confocal microscopy images of WT HeLa and μ1A KO cells stably expressing the streptavidin-KDEL hook transfected with either WT mCherry-APP-GFP or APP Y682A point mutation. Cells were imaged either 30 min (*top half of panels*) or 120 min (*bottom half of panels*) after addition of biotin to induce ER export. *A*–*F*, cells immunolabeled with anti-GM130. *H*–*M*, cells immunolabeled with anti-HRS. *A*, *D*, *H*, and *K*, WT HeLa cells expressing WT APP. *B*, *E*, *I*, and *L*, μ1A KO cells expressing WT APP. *C*, *F*, *J*, and *M*, WT cells expressing APP Y682A. Note the increased association of APP with Golgi marker GM130 and decreased association with endosomal marker HRS in both μ1A KO cells and with Y682A mutation after 120 min. *G*, APP co-localization with GM130. *N*, APP co-localization with HRS. Values represent mean ± SEM from at least seven different cells. *Main panels* scale bar represent 10 μm; *insets* (2×) scale bar represents 2.5 μm. ∗*p* ≤ 0.05; ∗∗*p* ≤ 0.01; and ∗∗∗*p* ≤ 0.001. Statistical significance was calculated by two-tailed Student's *t* test in *G* and *N*. APP, amyloid precursor protein; ER, endoplasmic reticulum.

The APP-RUSH results indicate a delay in anterograde transport of APP in cells lacking AP-1. To confirm this observation, we repeated these experiments and stained cells with either Golgi (GM130) or early endosome (HRS) markers by immunofluorescence. In these experiments, we monitored the GFP-containing molecules only, since they represent either full-length APP or APP CTFs. These findings show that whilst APP reaches the Golgi with similar efficiency in both WT and μ1A KO cells, the lack of AP-1 causes a clear delay in APP/APP-CTFs Golgi export (Fig. 7, *A*, *B*, *D* and *E*). This phenotype is accompanied by more efficient delivery of APP to early endosomes in WT compared with μ1A KO cells (Fig. 7, *H*, *I*, *K* and *L*). We also analyzed the anterograde transport of APP mutant Y682A and its co-distribution with either Golgi or early endosome markers using the RUSH system. The distribution pattern of APP Y682A in control cells was similar to that of WT APP in μ1A KO cells (Fig. 7, *C*, *F*, *J* and *M*). Together, these results show that either the absence of μ1A or the disruption of APP-μ1A interaction delays APP/CTF Golgi export and its delivery to early endosomes (Fig. 7, *G* and *N*).

### AP-1-mediated transport of APP affects the production and intracellular accumulation of C99

**Figure 8:**
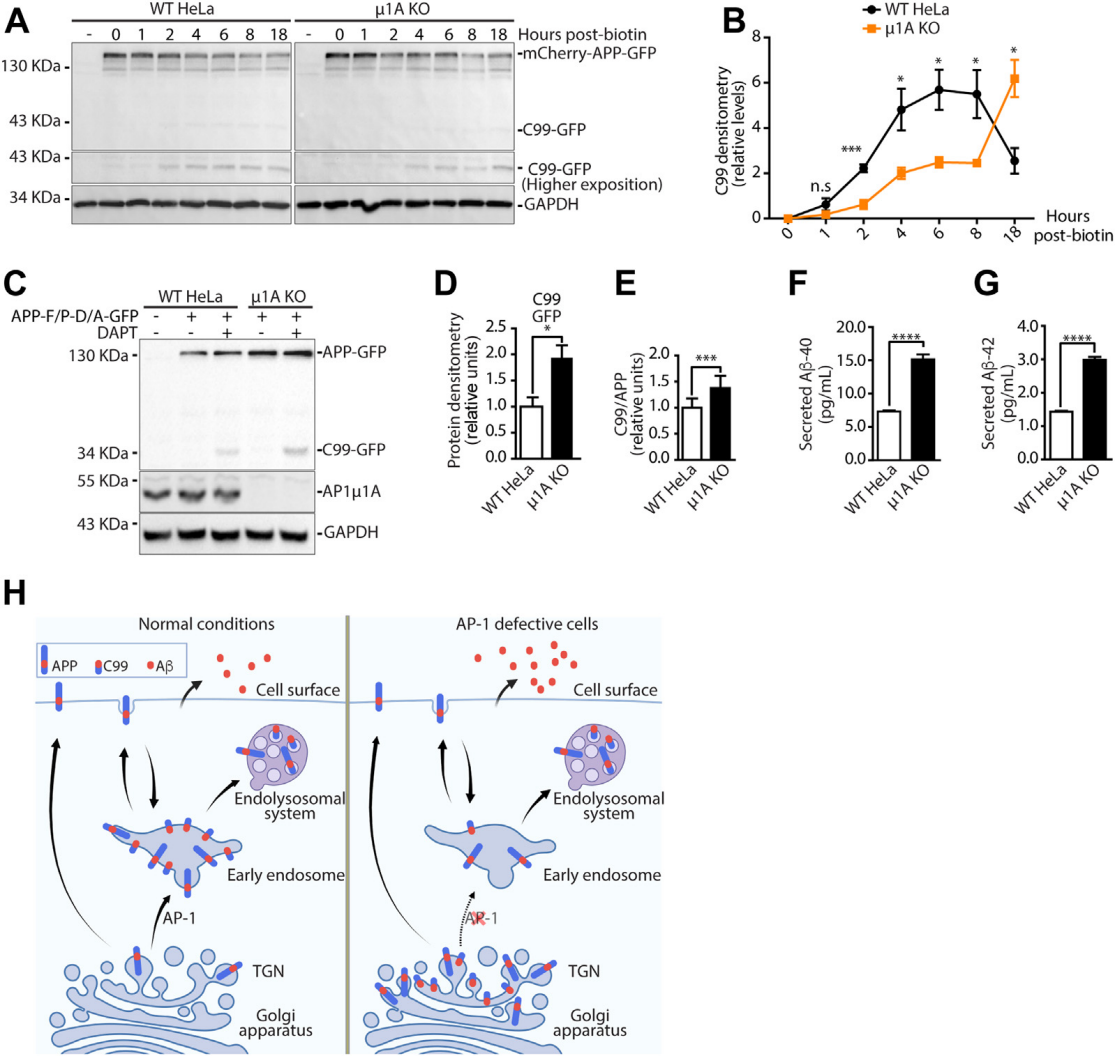
**AP-1 accelerates processing of APP to C99 and contributes to C99 clearance.***A*, Western blots of APP processing in WT and μ1A KO cells expressing streptavidin-KDEL, transfected with mCherry-APP-F615P/D664A-GFP, and incubated with 1 μM DAPT for 16 h. ER export was induced for 0 to 18 h. Membrane probed using 6E10 antibody specific to amyloid-β (as indicated for Fig. 3*A*). *B*, quantification of C99 levels in (*A*). *C*, Western blot of WT HeLa and μ1A KO cells transfected with APP-GFP F615P/D664A and incubated with or without 1 μM DAPT. Membranes probed using an anti-amyloid-β antibody. *D*, quantification of C99 levels in (*C*). *E*, the ratio of C99/APP in WT and μ1A KO cells from (*C*). APP and C99 levels were measured by band densitometry using Fiji software. Values represent mean ± SEM from at least three independent experiments. ∗*p* ≤ 0.05; ∗∗*p* ≤ 0.01; and ∗∗∗*p* ≤ 0.001. Statistical significance was calculated by two-tailed paired Student's *t* test in *B*, *D*, and *E*. *F* and *G*, ELISA showing the increased levels of Aβ1-40 (*F*) and Aβ1-42 (*G*) in the conditioned media of μ1A KO HeLa compared with WT cells, both expressing APP-GFP. A commercially purchased ELISA kit was used. Values represent mean ± SEM from six independent experiments. ∗∗∗∗ *p* ≤ 0.0001. Statistical significance was calculated by a two-tailed paired Student's *t* test. *H*, proposed model for AP-1-mediated APP trafficking. APP is synthesized in the ER and transported to the Golgi complex and then the TGN. In normal conditions, APP is efficiently sorted from the TGN to early endosomes by AP-1, where APP processing with C99 generation mainly occurs. In the absence of functional AP-1 (*right panel*), transport of APP from the TGN to early endosomes is delayed, increasing APP levels at the TGN. Prolonged retention results in C99 accumulation at the TGN. The delay of APP and C99 in exiting the TGN also reduces delivery to the endolysosomal system for clearance. This results in intracellular build-up of APP and C99 but with an increased ratio of the pathogenic fragment. AP-1, adaptor protein 1; APP, amyloid precursor protein; ER, endoplasmic reticulum; TGN, *trans*-Golgi network.

APP processing depends on its association with at least three secretase proteins in the endomembrane system (6). If β-secretase initiates the process, APP undergoes amyloidogenic processing, which requires γ-secretase activity to release the Aβ peptides (Fig. 3*A*). On the other hand, if APP processing is initiated by α-secretase, APP is directed to a non-amyloidogenic pathway and, after γ-secretase processing, a short peptide called P3 is released (Fig. 3*A*). To investigate how AP-1-mediated trafficking of APP influences processing, we monitored APP cleavage by Western blot using the RUSH system. WT HeLa and μ1A KO cells constitutively expressing streptavidin-KDEL were transfected with a RUSH mCherry-APP-F615P/D664A-GFP construct. These mutations in the APP CT inhibit α-secretase and caspase cleavage, favoring the visualization of the C99 fragment (41, 42, 43, 44). Moreover, the experiments were performed in the presence of a γ-secretase inhibitor, DAPT, to avoid further processing of C99 and allow its detection (10, 28, 44, 45) (Fig. 8*A*). At 20 h post-transfection, cells were incubated with soluble biotin for 0 to 18 h, lysed, and analyzed by Western blotting with a region-specific antibody (clone 6E10, indicated in Fig. 3*A*) to monitor C99 levels (Fig. 8, *A* and *B*).

The kinetics of C99 fragment generation were slower in the μ1A KO compared with WT cells, suggesting that β-secretase cleavage is less efficient in the Golgi than in endosomes. Despite this, 18 h after ER release, C99 accumulated intracellularly at higher levels in μ1A KO compared with WT cells (Fig. 8, *A* and *B*). In addition, we analyzed the processing of APP at steady state in WT HeLa and μ1A KO cells, with and without DAPT treatment, using APP-F615P/D664A-GFP. Compared with WT cells, AP-1-defective cells showed increased levels of both APP and C99 (Fig. 8, *C* and *D*) with an increased ratio of this pathogenic fragment (Fig. 8*E*), as observed for APP in the RUSH system at 18 h after ER release (Fig. 8, *A* and *B*). This indicates that APP transport mediated by AP-1 controls the processing of APP and intracellular levels of C99. Finally, to test whether the steady-state build-up of C99 changes amyloid-β production, we monitored the levels of amyloid-β secretion in the culture media of WT HeLa and μ1A KO cells expressing APP-GFP, using commercially available ELISA assays. The results show that AP-1-defective cells release higher levels of both Aβ-40 and Aβ-42 fragments compared with WT HeLa cells (Fig. 8, *F* and *G*).

Our findings suggest that AP-1 is essential to mediate the efficient transport of APP and C99 from the Golgi to endosomes, a crucial step in lysosomal-mediated intracellular clearance of pathogenic C99, the direct precursor of amyloid-β (Fig. 8*H*).

## Discussion

Deciphering the molecular mechanism regulating APP trafficking is of great interest as its amyloidogenic processing is a major causative factor of AD. Evidence shows that C99 fragments accumulate in the hippocampus of AD mouse models during early pathological stages (46), and it is thought to contribute to synaptic plasticity impairments (25, 47, 48). It has been suggested that C99, rather than Aβ plaques, is responsible for neuronal death in AD (49). Here, we showed that the clathrin adaptor AP-1A is an essential factor controlling the distribution of APP and C99 within the secretory pathway. Our data show that AP-1A binds the APP/C99 CT at the TGN and mediates transport from the TGN to the endolysosomal system. Disrupting this AP-1-mediated transport route causes the intracellular build-up of C99, most likely because of impaired Golgi exit and endosomal delivery, leading to the reduced clearance of C99 (Fig. 8*H*).

APP is known to interact with both AP-1A and the epithelial cell–specific variant AP-1B (21). However, knowledge about the functional relevance of these interactions was restricted to AP-1B, which was implicated in the delivery of APP to the basolateral domain of polarized epithelial cells (21). To understand the functional role of AP-1A-mediating APP transport through the secretory pathway, we used synchronized trafficking assays. We show that the APP tail contains information required for its efficient anterograde transport and mapped Y682 as a crucial residue mediating this process (Figs. 1 and 2). We also show that Y682 is essential for the interaction with both AP-1A and AP-1B (Figs. 4 and S3). This tyrosine residue is part of the _682_YENPTY_687_ domain, a well-characterized sorting signal in APP (6). The specific analysis of APP Y682 was important because this tyrosine residue is not required for APP interaction with the μ4 subunit of AP-4 (10). Therefore, this mutant enabled us to investigate APP transport dependent on AP-1 interaction, without disturbing APP interaction with AP-4. However, mutations in _687_YKFFE_691_ abolished the interaction of APP with μ1A, μ1B, and μ4 (Fig. S3), indicating that AP-1 and AP-4 bind to common residues within this APP region.

We further demonstrated that APP and TGN38 interactions with AP-1 involve two structurally separate domains in μ1A, termed A-site and B-site (Fig. 4). Consistently, previous work showed the participation of two recognition sites in μ1A for interaction with the CT of the viral glycoprotein NiV-F (37). The distance between the two binding sites in μ1A is 30 Å, and an unstructured peptide composed of 14 amino acids is approximately 45 Å long (37). Considering that the APP CT has 47 amino acid residues and the TGN38 CT has 33 residues, simultaneous interaction with both binding sites in μ1A should be physically possible. Previous work reported a third domain in μ1A composed of basic amino acids that work together with the tyrosine-binding pocket to regulate major histocompatibility complex I molecules and Nef interaction (19, 50), also suggesting that stable μ1A interaction with AP-1 cargo relies on multiple interaction points.

A central finding from our study is that APP interaction with AP-1A is required for its efficient Golgi export, which was demonstrated using several complementary approaches. Initially, we used a quantitative approach to show that μ1A expression is essential for APP anterograde trafficking and processing (Fig. 4). Consistently, γ1 depletion by RNAi in H4 neuroglioma cells, a condition that also compromises the expression of μ1A (51, 52), redistributes endogenous APP from the cell periphery to the juxtanuclear region and increases its co-localization with a TGN marker (Fig. S4). A similar phenotype was also observed in HeLa μ1A KO cells expressing APP-GFP (Fig. 5). Importantly, redistribution of APP to the Golgi in the KO cells was reversed by expression of exogenous μ1A, confirming the specificity of the μ1A KO phenotype (Fig. 5).

Supporting our findings that AP-1A activity is required for normal APP trafficking, the over-expression of a μ1A mutant that does not bind APP (μ1A W408S), but remains capable of forming an AP-1 complex (20, 40), increased the association of APP with TGN46 in H4 cells (Fig. 6). The expression of this dominant-negative version of μ1A has been used in several studies to identify AP-1 cargo proteins and study their transport mediated by AP-1 (20, 37, 40). Consistent with the results in H4 cells, expression of μ1A W408S in primary cultured neurons also increased the co-localization of APP with a Golgi marker in the cell body (Fig. 6).

Evidence for the role of AP-1 in APP trafficking was further strengthened by comparing the subcellular distribution of WT APP and the APP Y682A mutant that is unable to bind AP-1 while preserving AP-4-binding capacity. In both primary neurons and H4 cells, the amount of APP Y682A mutant retained in the Golgi/TGN is higher compared with WT APP (Fig. 3). Moreover, APP Y682A is enriched at the cell surface, displays less association with early endosomal markers, but shows an increased association with late endosomal markers (Figs. 3 and S2). While these observations are consistent with previous reports (24, 53, 54, 55, 56), none of these previous studies have correlated the function of AP-1 with changes in APP localization. A possible interpretation of these findings is that AP-1 mediates an efficient direct route of APP transport from Golgi to early endosomes, but in the absence of AP-1 interaction, APP may follow the constitutive secretory pathway, or an AP-4 mediated route (10), to the cell surface and/or late endosomes.

It is well established that AP-1 mediates transport between the TGN and endosomes, with evidence for participation in both anterograde and retrograde transport routes depending on the cargo (14). When AP-1 activity is perturbed, we observe an accumulation of APP at the TGN during steady state (Figs. 3, 5 and 6). Despite this, these experiments did not allow us to distinguish between a defective anterograde transport from the TGN and accelerated retrieval from endosomes. Therefore, to define the transport route mediated by AP-1 in APP transport, we used the RUSH system (22) to monitor the anterograde transport of APP in a synchronized fashion (Fig. S5). Using this approach, we show that the efficient exit of APP from the Golgi and its delivery to early endosomes is impaired when the APP–AP1A interaction is disrupted. Similar results were shown by either mutating the interaction motif in APP or depleting cells of μ1A (Fig. 7).

Retention of APP in the Golgi/TGN was previously shown to promote Aβ secretion and increase the intracellular levels of C99, upon inhibition of γ-secretase activity (10, 11, 12). It was proposed that BACE1-mediated cleavage of APP is enhanced when APP export from the TGN is blocked (11). Consistently, we found that impaired Golgi/TGN export because of AP-1 depletion increased the intracellular levels of C99, and consequent Aβ-40 and Aβ-42 release, at steady state (Fig. 8). Despite this, we have previously shown that autophagosomes are critical organelles in the turnover of APP and its CTFs and that this process involves their transport to the endolysosomal system (41). This could provide an alternative explanation for the increase in C99 levels when Golgi export is defective, impairing delivery to the endolysosomal system. Using the synchronized RUSH transport assay, we can distinguish between increased BACE-1 processing from impaired C99 turnover, and our data support the latter hypothesis (Fig. 8).

Collectively, our results define AP-1A as key sorting machinery directly controlling the subcellular distribution of APP, with a decisive role in transporting APP/C99 from the TGN to the endolysosomal system, contributing to pathogenic C99 fragment clearance (Fig. 8*F*). Therefore, defects in AP-1-mediated transport of APP/C99 can be regarded as a potential contributing factor to AD etiology.

## Experimental procedures

### Plasmids for RUSH assays

#### Streptavidin-KDEL

A streptavidin-KDEL construct was generated for use as the ER hook in the RUSH system. The streptavidin gene (generous gift from Juan S. Bonifacino; National Institute of Child Health and Human Development, National Institutes of Health (57)) was subcloned into the Clontech pQCXIP Retroviral Vector by Gibson Assembly, using the following primers:

Forward primer: CCAAC TTTCC GTACC ACTTC CTACC CTCGT AAAGG CCACC ATGGA TGTAT GCGTC CGTCT TGCCC TGTGG

Reverse primer: GAGGG GCGGA ATTCG GATCC TATCT CGAGA TCACA GTTCA TCTTT CAGAT CCTCT TCAGA GATGA GTTTC TGTTC CGGTC CGAGC TGCTG GACGG CATCC AGAGG

#### Halo-APPwt-mNeonGreen

To generate the Halo-APP-mNeonGreen RUSH constructs, DNA fragments for the signal peptide (SP) and SBP (generous gift from Juan S. Bonifacino (57)) were subcloned into the Clontech vector eGFP-C1, to generate an SP-SBP-GFP-LAMP1_delYQTI construct. A HaloTag was then subcloned into the N terminus of LAMP1 in place of the enhanced GFP tag by Gibson assembly. This generated an SP-SBP-HaloTag-LAMP1_delYQTI construct. To do this, the following primers were used:

Backbone forward primer: CTGTC CACGC TCGAG ATTTC CGGCC CGGCC AGACG CCCCA GCACT G

Backbone reverse primer: GGAAA GCCAG TACCG ATTTC CATTG CAGGT GGTTC ACGTT GACCT TG

Insert forward primer: CAAGG TCAAC GTGAA CCACC TGCAA TGGAA ATCGG TACTG GCTTT CC

Insert reverse primer: CAGTG CTGGG GCGTC TGGCC GGGCC GGAAA TCTCG AGCGT GGACA G

APP (kindly donated by Juan Bonifacino) and mNeonGreen (Integrated DNA Technologies, Inc) gene fragments were subcloned into the C terminus of the HaloTag by Gibson assembly, using the following primers:

APP forward primer: CGCGC GCTGG CTGTC CACGC TCGAG ATTTC CGGCG GCGGC AGCCC CACTG ATGGT AATGC TGGCC

APP reverse primer: ATATT ATCTT CTTCG CCCTT GCTAA CCATG CTGCC GCCGT TCTGC ATCTG CTCAA AGAAC

mNeonGreen forward primer: GTTCT TTGAG CAGAT GCAGA ACGGC GGCAG CATGG TTAGC AAGGG CGAAG AAGAT AATAT

mNeonGreen reverse primer: GATTA TGATC AGTTA TCTAG ATCCG GTGGA TCCTA TTTAT AAAGC TCGTC CATGC CCATA ACATC CG

The SP-SBP-HaloTag-LAMP1_delYQTI backbone was digested with XhoI and BamHI to excise the LAMP1 gene fragment, generating an SP-SBP-HaloTag-APP-mNeonGreen construct.

Subsequently, the HaloTag-APP-mNeonGreen gene fragment was amplified by PCR using the following primers:

Forward primer: CATTT TGGCA AAGAA TTGTG TACAA GGATC CGCTA GCGCT ACGCG C

Reverse primer: GCCTG CACCT GAGGA GTGAA TTCAC GCGTG GATCC TATTT ATAAA GCTCG TCCAT GCC

The PiggyBac Transposon backbone (a kind gift from Michael Ward; National Institute of Neurological Disorders and Stroke, National Institutes of Health) was digested using MluI and BsrGI, and the Halo-APP-mNeonGreen gene fragment was inserted by Gibson assembly. This construct was used to generate stable Halo-APP-mNeonGreen cell lines by co-transfection with a transposase plasmid (kindly donated by Michael Ward).

#### Halo-APPmut-mNeonGreen

PCR primers were designed to introduce point mutations into the CT of APP. Halo-APPwt-mNeonGreen was amplified by PCR using mutation-specific primers, and a KLD reaction was carried out (NEB; catalog no.: M0554S). Sanger sequencing was used to confirm the presence of the correct mutation in each construct.

#### Transient CRISPR KO plasmids

To generate transient CRISPR KOs of μ1A and μ1B, the IDT Alt-R CRISPR/Cas9 guide RNA tool was used to design two custom-guide sequences per gene. These guides were cloned into a pKLV-U6gRNA(BbsI)-PGKzeocin2ABFP vector, using the BbsI restriction sites. A Cas9 viral expression backbone and the packaging vectors pMD.G and pCMVR8.91 were kindly gifted to us by Paul Lehner (University of Cambridge).

#### Monocistronic mCherry-APP-GFP

The mCherry-APP-GFP RUSH monocistronic vector comprises a cyctomegalovirus promoter to express APP fused to the IL-2 signal sequence, SBP, and mCherry at the N terminus, and GFP at the C terminus. The complementary DNA fragment comprising IL-2-SBP-mCherry-APP from bicistronic mCherry-APP-GFP was subcloned into the commercial plasmid pEGFP-C2 (Clontech) upstream of the GFP coding sequence. The APP sequence contains two-point mutations, F615P and D664A, inserted by site-directed mutagenesis (QuickChange—Agilent). Sanger sequencing was used to confirm the presence of the correct mutation in each construct.

### Other plasmids

The plasmids containing the full-length sequences of mouse μ1A (including D174A and W408S mutants), mouse μ2, rat μ3A, human μ4, and the C-terminal domain of human μ1B (residues 137–423), subcloned in the pACT2 vector in fusion with the Gal4-activation domain (Clontech) have been described previously (19, 40). Mouse μ1A and W408S mutant fused in the C terminus with a 10-amino-acid linker and three copies of HA epitope in the pCI-neo vector (Clontech) were previously described (40). These plasmids were kindly donated by Juan Bonifacino. Human APP CT (isoform 695) and TGN38 CT were subcloned in fusion with Gal4-binding domain in the vectors pGBKT7 and pGBT9, respectively (10, 34). All point mutations in APP and μ1A subunits were generated by site-directed mutagenesis (QuickChange—Agilent) or by KLD (New England Biolabs) and confirmed by Sanger sequencing. APP containing the mutations F615P and D664A subcloned in pEGFP-N1 (Clontech) were previously described (10). APP F615P/D664A were then subcloned into pmCherry-N1 using XhoI and HindIII restriction sites.

### Antibodies

For immunofluorescence assays, the following antibodies were used: the monoclonal mouse antibodies to APP/amyloid-β (1:100 dilution; catalog no.: 803015; clone 6E10; BioLegend), γ1 adaptin (1:100 dilution; catalog no.: 610385; clone 88; BD Biosciences), anti-HA (1:200 dilution; catalog no.: H3663; Sigma), EEA1 (1:200 dilution; catalog no.: 612006; clone 14/EEA1; BD Biosciences), CD63 (1:200 dilution; catalog no.: 556019; clone H5C6; BD Biosciences), GM130 (1:200 dilution; catalog no.: 610822; clone 35/GM130; BD Biosciences), sheep polyclonal anti-TGN46 (1:400 dilution; catalog no.: AHP500; Bio-Rad), and rabbit polyclonal anti-HGS/HRS (1:200 dilution; catalog no.: AB155539; Abcam). Secondary antibodies conjugated to Alexa fluorophores were purchased from Thermo Fisher Scientific.

For immunoblotting assays, the following antibodies were used: monoclonal mouse anti-γ1 adaptin (1:1000 dilution; clone 88; BD Biosciences), rabbit polyclonal anti-μ1A-adaptin (1:1000 dilution; catalog no.: AB111135; Abcam), mouse monoclonal anti-HA (1:1000 dilution; Sigma), monoclonal mouse anti-actin-β (1:5000 dilution; catalog no.: MA1-91399; Thermo Fisher Scientific), and rabbit polyclonal anti-GAPDH (1:5000 dilution; catalog no.: G9545; Sigma). Rabbit polyclonal anti-GFP (1:5000 dilution) was a gift from R. Hegde (MRC) and was previously used in monoclonal mouse anti-APP/amyloid-β (1:1000 dilution; catalog no.: 803015; clone 6E10; BioLegend), rabbit anti-amyloid-β polyclonal antibody (1:1000 dilution; catalog no.: 51-2700; Thermo Fisher Scientific), mouse anti-amyloid-β antibody clone W0-2 (1:1000 dilution; catalog no.: MABN10; Merck), rabbit anti-GAPDH horseradish peroxidase (HRP) conjugate antibody (D16H11) (1:1000 dilution; catalog no.: 8884; Cell Signaling Technology), rabbit anti-AP1μ1A (1:1000 dilution; catalog no: 12112-1-AP; ProteinTech), and rabbit anti-AP1μ1B (1:1000 dilution; catalog no.: 10618-1-AP; ProteinTech) (58). Either HRP-conjugated donkey anti-mouse and donkey anti-rabbit (GE Healthcare) or goat HRP-conjugated secondary antibodies (1:5000 dilution; Abcam) were used for immunoblotting.

### Cell culture, transfections, and RNA interference

Stable CRISPR/Cas9 μ1A KO and WT HeLa cells were a gift from Margaret S. Robinson (University of Cambridge) and have been previously described (20). H4 (human neuroglioma) cells were obtained from the American Type Culture Collection. PEAK cells are HEK-293T cells transfected with the large T antigen of SV-40 (59) and were used for co-immunoprecipitation experiments. To generate HeLa cell lines expressing ER-targeted streptavidin fused to KDEL (ER hook), retrovirus coding streptavidin-KDEL was produced in HEK-293T cells and used to transduce HeLa cells. Transduced cells were selected by incubation in complete media in the presence of 1 μg/ml puromycin. Cells were maintained as previously described (60). DNA transfections were performed using Lipofectamine 2000 (Thermo Fisher Scientific). The siRNAs were purchased from Dharmacon as nucleotide duplexes with 3′dTdT overhangs, designed to target human γ1 (5′-GGAAGAGCCUAUUCAGGUA-3′) (51). Transfections of siRNA were performed in two rounds with an interval of 48 h between treatments using Oligofectamine reagent (Thermo Fisher Scientific).

### Mammalian cell culture for the APP mutant screen

HeLa cells were cultured in Dulbecco’s modified Eagle's medium (DMEM) (Sigma; catalog no.: D6429) supplemented with 1% MycoZap and 10% fetal calf serum at 37 °C with 5% CO_2_. Where puromycin resistance was conferred by stably expressing streptavidin-KDEL, the ER hook, HeLa cells were cultured in 1 μg/ml puromycin. Streptavidin-KDEL cells were further engineered to stably express Halo-APPwt-mNeonGreen using the PiggyBac system. To do this, streptavidin-KDEL HeLa cells were plated at 60% confluency in a 6-well plate and adhered overnight. Cells were then transfected with both a transposase and transposon plasmid using Lipofectamine 2000 (Thermo Fisher Scientific; catalog no.: 11668019). The ratio of Lipofectamine 2000 (micoliter) to DNA (microgram) was 2.5. To transfect one well of a 6-well plate, 4 μg DNA was diluted in 200 μl Opti-MEM I Reduced Serum Medium and incubated for 5 min at room temperature (RT). 10 μl Lipofectamine 2000 was diluted in 200 μl Opti-MEM I and incubated for 5 min at RT. Solutions were mixed and incubated for 20 min before dropwise addition to the cells. The ratio of transposase to transposon was 2:1. After 3 days, 0.25 mg/ml hygromycin was added to cells. After 1 week, cells were split into 96-well plates at a concentration of one cell per well to obtain clonal cell lines.

### Transient CRISPR KO cells

To generate a stable Cas9-expressing Halo-APP-mNeonGreen cell line, HeLa cells stably expressing streptavidin-KDEL and Halo-APP-mNeonGreen were infected with lentiviral particles carrying Cas9 plasmid DNA (generous gift from Paul Lehner, University of Cambridge), followed by selection in 150 mg/ml blasticidin (Gibco). Lenti-XTM 293T cells were used to package the pKLV-zeocin vectors encoding the guide RNAs into lentiviral particles. After 48 h, the viral supernatants were harvested, filtered using a 0.45 μM filter, and concentrated to 10× using a Lenti-X Concentrator (catalog no.: 631232; Takara Bio). To achieve transient AP1μ1A and μ1B knockouts, approximately 25 × 10^5^ Cas9 Halo-APP-mNeonGreen cells were transduced with 30 μl of 10× concentrated lentiviral particles in a 48-well plate. After 48 h, cells were replated into a 6-well plate and incubated for 4 days. On day 6, RUSH assays were carried out and analyzed by flow cytometry, as described later. Depletion of AP1μ1A and μ1B was verified by immunoblotting.

### RUSH assays

For flow cytometry, HeLa cells stably expressing both streptavidin-KDEL and Halo-APPwt-mNeonGreen were lifted with trypsin, pelleted at 500 relative centrifugal force (RCF) for 5 min, and washed in PBS (CaMg). The cells were resuspended in CO_2_-independent media (DMEM + 25 mM Hepes) and aliquoted into eppendorfs at 500,000 cells in 500 μl. For non-perturbed RUSH, eppendorfs were incubated at 37 °C in a heat block (DB200/2; Techne). A solution of 2× D-biotin (B4501; Sigma–Aldrich) in DMEM (500 μM final concentration) was added to each eppendorf in turn to generate ER export times of 0 to 5 h. In the last 30 min, 2× Halo-JF646 HaloTag ligand was added to each sample. Protein trafficking was stopped by transferring samples to ice, followed by centrifugation (500 RCF, 4 °C, 5 min). Samples were resuspended in 500 μl ice-cold PBS and filtered using Cell-Strainer-capped 5-ml round-bottom tubes (catalog no.: 352235; Corning). HeLa cells stably expressing the streptavidin-KDEL hook but no APP construct were used as negative controls (with/without biotin). A minimum of 30,000 cells per sample were analyzed using an LSRFortessa cell analyzer (BD Biosciences), gating for mNeonGreen and Halo-JF646-positive cells. Data were analyzed using FlowJo software (FlowJo Software, version 10.7.1, for Mac OS X, Becton Dickinson & Company [BD] 2006–2020).

For 20 °C block experiments, the aforementioned protocol was carried out at 20 °C (Fig. S1). Several molecular inhibitors were used in combination with the protocol described previously (Fig. 1). The concentration and length of inhibitor treatment varied depending on the recommended conditions or those used previously in the literature. The concentration of each inhibitor was maintained during the 5 h RUSH. The following pre-treatments were used: BFA: 10 μg/ml BFA for 1 h prior to inducing ER export; DAPT: 25 μM DAPT for 24 h prior to inducing ER export and MG132: 10 μM MG132 for 2 h prior to inducing ER export.

To prepare samples for immunofluorescence microscopy, cells were transfected with a bicistronic mCherry-APP-GFP RUSH plasmid and incubated for 4 h, followed by an additional 16 h incubation, as described in the figure legend. Cells were then treated with 40 μM final concentration of soluble biotin (Sigma–Aldrich) and incubated for different time points, after which cells were fixed for immunofluorescence microscopy.

To prepare samples for Western blots, a HeLa cell line expressing ER-targeted streptavidin-KDEL were transfected with a monocistronic mCherry-APP-GFP RUSH plasmid and incubated for 4 h, followed by an additional 16 h in the presence of 1 μM DAPT, as described in the figure legends. Cells were then treated with 40 μM final concentration of soluble biotin (Sigma-Aldrich) and incubated in the presence of 1 μM DAPT for different time points, after which cells were lysed for Western blot.

### Sequence alignment and protein model images

The sequence of CTs of APP homologous proteins from different species and the human APP gene family were obtained from the National Center for Biotechnology Information and aligned using the free software Clustal Omega alignment (61). Surface representation of μ1A C-terminal domain was collected from Protein Data Bank (1W63) (62). Protein model manipulation was performed using PyMOL (www.pymol.org).

### Y2H assays

Y2H assays were performed using the yeast AH109 reporter strain as described previously (63). Yeast containing both plasmids with GAL4 activation domain and binding domain were selected in a restriction medium without leucine and tryptophan. Protein interactions were visualized by yeast growth in a selective medium lacking histidine, leucine, and tryptophan.

### GFP-trap assay

GFP-Trap agarose (ChromoTek) assay was performed following the manufacturer's recommendations. Briefly, 80% confluent PEAK cells placed in a 100 mm plate were transfected with plasmids to express GFP, APP-GFP, or C99-GFP with μ1A-HA. After overnight expression, cells were lysed with lysis buffer containing 10 mM Tris–HCl (pH 7.5), 150 mM NaCl, 0.5 mM EDTA, and 0.5% Nonidet P40. The cleared protein content was incubated with beads for 1 h at 4 °C under rotation. Next, beads with the binding proteins were washed three times with wash buffer (10 mM Tris–HCl [pH 7.5], 150 mM NaCl, and 0.5 mM EDTA), followed by the analysis of the attached protein by immunoblotting.

### Immunodot blot assay

HeLa cells stably expressing both streptavidin-KDEL and Halo-APPwt-mNeonGreen were seeded at 40% confluency in a 6-well plate 24 h before inducing ER export. On the following day, cells were stained with JF646 HaloTag ligand for 1 h and washed twice in PBS (CaMg) to remove unbound JF646 dye. The media were aspirated from one well and replaced with complete DMEM + 500 μM biotin every hour for 5 h, to induce ER export for 0 to 5 h. Subsequently, the media were removed from each well and centrifuged at 500 RCF for 5 min, followed by a second centrifugation at 17,000 RCF for 5 min in a 4 °C centrifuge. 700 μl of each sample was spun in a 3 kDa amicon column, and a media exchange was carried out so that the protein was suspended in approximately 90 μl PBS (CaMg). Each sample was then made up to 100 μl total with PBS (CaMg). 1 μl (1%) of each sample was added in a line to nitrocellulose membrane. The membrane was left to dry for 30 min before it was soaked in PBS (CaMg) and sealed between two acetate sheets. The membrane was then imaged using a BioRad ChemiDoc Imaging System.

### Fluorescence plate reader assay

HeLa cells stably expressing both the streptavidin-KDEL hook and Halo-APPwt-mNeonGreen were trypsinised and washed twice in PBS (CaMg). Cells were incubated in imaging media (DMEM without phenol red + 2% bovine serum albumin + 4 mM l-glutamine + 25 mM Hepes) + JFX650 HaloTag ligand for 30 min at 37 °C. After this time, cells were washed twice with imaging media (5 min/wash at 37 °C) to remove the unbound HaloTag. Subsequently, cells were resuspended in imaging media, aliquoted into eppendorfs, and incubated at 37 °C. Every hour, 2× biotin (500 μM final concentration) in imaging media was added to the corresponding tube to induce ER export. Eppendorfs were vortexed every 30 min. After 5 h, protein trafficking was halted by transferring samples to ice, followed by centrifugation (500 RCF; 5 min; 4 °C). The supernatant was removed from cells and kept on ice. The cell pellet was washed in PBS (CaMg) before lysis in 500 μl radioimmunoprecipitation buffer + 1 μl benzonase for 30 min on ice. After lysis, the media samples and lysates were centrifuged at 17,000 RCF for 15 min at 4 °C. The supernatants were collected from samples and 200 μl of each was loaded into a 96-well imaging plate (PerkinElmer). Two bovine serum albumin standards, diluted in either cell medium or lysis buffer, were loaded alongside the samples to measure Halo-JFX650 saturation levels. Samples were quantified in a CLARIOstar *Plus* plate reader (BMG Labtech).

### SDS-PAGE and immunoblot analysis

SDS-PAGE and immunoblotting analysis were performed as previously described (58). In brief, denatured cell extracts from 200,000 cells were resolved on either gradient Tris–Glycine acrylamide gels (Bio-Rad) or home-made 10 to 16% Tris–Glycine acrylamide gels. Proteins were transferred to a polyvinylidene difluoride membrane using a wet transfer protocol and blocked with 5% skimmed milk for 1 h. Membranes were incubated with the appropriate primary antibody overnight at 4 °C, washed with PBS with Tween-20, and then incubated with a secondary antibody for 1 h at RT. The membranes were visualized using either Clarity (catalog no.: 1705061; Bio-Rad) or WesternBright Sirius (catalog no.: K-12043-D10; Advansta) enhanced chemiluminescence solutions and a ChemiDoc Imaging System equipped with the ImageLab software (Bio-Rad Laboratories).

### Primary neuron culture and transfection

Primary neuronal cultures were isolated from embryonic day 18 Wistar rats using the same protocol as previously described (64). Briefly, cortical areas were dissected, subjected to digestion with trypsin (Sigma–Aldrich), and mechanically dissociated with DNAse (Sigma–Aldrich). Cells were plated onto 22 mm glass coverslips coated with poly-l-lysine hydrobromide (0.5 mg/ml; Sigma–Aldrich). The plating medium consisted of Neurobasal medium (Gibco) supplemented with 1% penicillin–streptomycin (Invitrogen), 0.5% l-glutamine (Formedium), 2% B-27 (Gibco), and 5% horse serum (Invitrogen). The following day, the plating medium was changed for horse serum–free feeding medium. Cultures were maintained at 37 °C and 5% CO_2_ in a humidified incubator. Transfection was performed using Lipofectamine 2000 (Life Technologies) and cortical neurons at 12 days *in vitro* (65). Immunocytochemistry assays were performed 16 h after transfection, as previously described (65). The animals were treated in accordance with the Animal Welfare and Ethics Review Body Committee, and experiments were performed under the appropriated project licenses with local and national ethical approval. All experiments involving animals have been designed in consideration of the guidance provided by NC3Rs (nc3rs.org.uk).

### Fluorescence microscopy for Figure 1

For fixed cell imaging, cells were seeded on Matrigel-coated glass coverslips. The Matrigel coating solution consisted of 500 μl concentrated Matrigel (Corning; catalog no.: 354277) in 50 ml DMEM/F12 medium (Gibco). 1 ml/well Matrigel solution was added to coverslips in 6-well plates and incubated for 24 h before cells were seeded. HeLa cells stably expressing Halo-APP-mNeonGreen and streptavidin-KDEL were seeded at 100,000 cells/well on Matrigel-coated glass coverslips 24 h before biotin addition. Subsequently, media were removed from each well in turn and replaced with 2 ml complete DMEM + 500 μM biotin/well to generate ER export times of 0 to 5 h. After 5 h, cells were fixed in a cytoskeleton fixation buffer (300 nM NaCl, 10 mM EGTA, 10 mM glucose, 10 mM MgCl_2_, 20 mM Pipes [pH 6.8], 4% paraformaldehyde [PFA], and 2% sucrose) for 10 min at RT, before washing twice in PBS (supplemented with 100 mg/l calcium chloride and 100 mg/l magnesium chloride ions [Gibco]) and once in water. Coverslips were mounted in ProLong Gold Antifade Mountant containing 4′,6-diamidino-2-phenylindole to visualize the nucleus (Invitrogen).

### Immunofluorescence microscopy

Immunofluorescence was performed as previously described with no modifications (51). Briefly, cells were fixed for 15 min at RT with 4% (w/v) PFA in PBS. PFA-fixed cells were permeabilized with 0.01% (w/v) saponin in blocking solution (0.2% [w/v] pork skin gelatin in PBS) for 30 min at 37 °C, and double labeled with specific primary and secondary Alexa-conjugated antibodies. Cells were imaged on a Zeiss confocal laser-scanning microscope 780 (Zeiss). Post-acquisition image processing was performed with Fiji/ImageJ software (https://imagej.net/software/fiji/) (66). Co-localization analysis was performed using the Fiji/ImageJ plugin co-localization threshold to determine the Pearson's correlation coefficient among two channels, using Z-stacks with 0.3 μm intervals of at least seven cells from three independent experiments. Pearson's correlation coefficient represents all nonzero-zero pixels that overlay the images of the channels and returns a value between −1 and +1, where +1 is a total positive correlation, 0 is no correlation, and −1 is a total negative correlation.

### Statistical analysis

For fluorescence-activated cell sorting analyses, the mean of a minimum of 30,000 cells per repeat was standardized as a percentage of the 0 h measurement and, since the measured fluorescent levels are expected to decay exponentially over time, transformed by a natural logarithm (time points 1–5 h). We found a good fit for a linear decay after logarithm transformation. Statistical analysis with three repeats of each condition was performed using R with the lme4 package, and statistical significance was considered when *p* < 0.05. Separately for each baseline/condition (*i.e.*, either CRISPR KO or mutation) combination, a random intercept model from linear mixed-effect regression was employed to model the change in fluorescence intensity. The fixed effects included in the model were (a) the overall mean intercept, (b) time of measurement, (c) whether the mutation was present, and (d) an interaction effect between the time of measurement and the presence of the mutation modifying the slope of the linear decay. In addition, we fitted a model with factors (a, b, and c) only and a third model with factors (a and b). The three models were compared by likelihood-ratio tests. When comparing the first and second models, a small *p* value indicates a significantly different slope for the condition curve from the baseline curve (“slope significance”), whereas when comparing the first and third models, a significant test result indicates that including any condition-level effects (intercept and slope) yields a better fit (“intercept significance”).

### For all other figures

Statistical data are demonstrated as mean ± SEM, and the n samples are indicated in the figure legend for each analysis. The statistical analysis to determine significance is described in each figure legend. The *p* values are labeled as follows: ∗*p* ≤ 0.05; ∗∗*p* ≤ 0.01; ∗∗∗*p* ≤ 0.001; and ∗∗∗∗*p* ≤ 0.0001. Differences were considered statistically significant at *p* ≤ 0.05. Data were plotted and analyzed using either GraphPad Prism 5.0 software (GraphPad Software by Dotmatics) or PyCharm CE (JetBrains).

### Aβ ELISAs

To determine the content of Aβ1-40 and Aβ1-42 fragments of APP in conditioned media, equal numbers of HeLa cells were mock transfected or transfected with a plasmid encoding APP-GFP using Lipofectamine 2000 and incubated for 20 h. After incubation, cells were washed three times with Opti-MEM and incubated for another 16 h in Opti-MEM. After incubation, conditioned media were collected, cleared from cell debris, and equalized for total protein content. The levels of secreted Aβ1-40 and Aβ1-42 were determined using commercially available kits (Thermo Fisher Scientific; catalog nos.: KHB3481 and KHB3544, respectively), according to the manufacturer's instructions. The ELISA data were from six independent experiments.

## Data availability

All data are included in the article and supporting information.

## Supporting information

This article contains supporting information.

## Conflict of interest

The authors declare that they have no conflicts of interest with the contents of this article.
