# Supplementary material for: Clathrin adaptor AP-1–mediated Golgi export of amyloid precursor protein is crucial for the production of neurotoxic amyloid fragments
Source: J Biol Chem. 2022 Jun 23;298(8):102172. doi: 10.1016/j.jbc.2022.102172 (PMC9352552; doi:10.1016/j.jbc.2022.102172)
Supplement: Supplemental Figures S1–S5 [file mmc1.pdf]

Clathrin adaptor AP-1-mediated Golgi export of amyloid precursor protein is crucial for the production of neurotoxic amyloid fragments.

Yunan C. Januário[1,2]*; Jessica Eden[3]*; Luan S. de Oliveira[1,2,4]; Raffaella De Pace[5]; Lucas A. Tavares[1,2]; Mara E. da Silva-Januário[1,2]; Vinícius B. Apolloni[1,2], Elise L. Wilby[3], Randolf Altmeyer[6], Patricia V Burgos[7,8]; Sonia A.L. Corrêa[4,9], David C. Gershlick[3]#; and Luis L. P. daSilva[1,2]#

Supporting Information

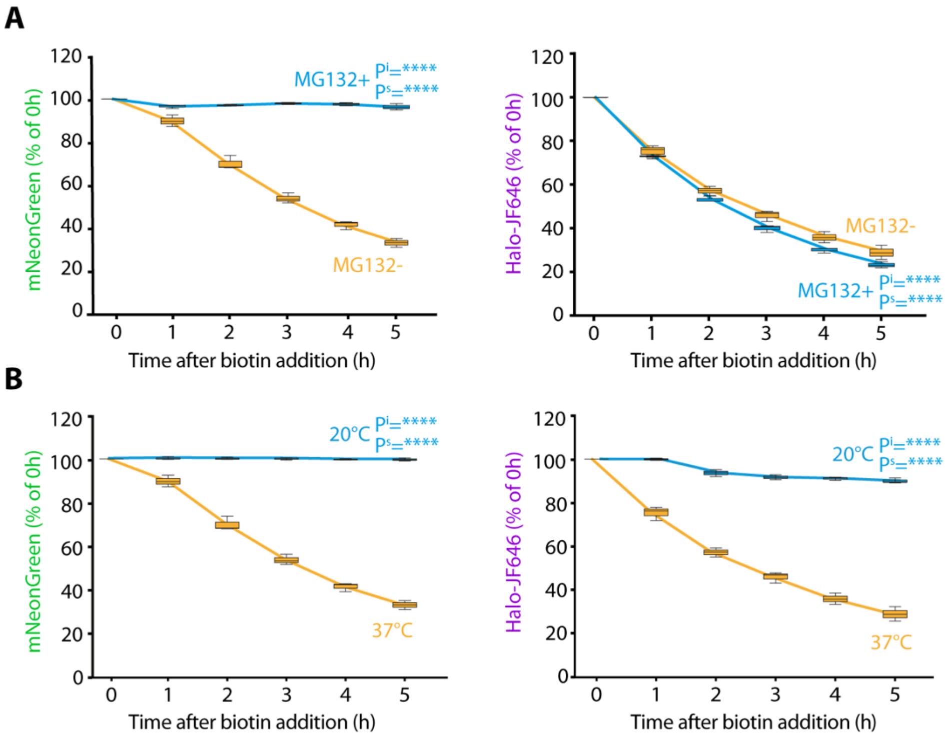

Supplementary Figure 1: Efficient Halo-APP-mNeonGreen processing takes place after Golgi export. (A) Halo-APP-mNeonGreen RUSH cells were pre-treated with 10 µM MG132, a proteasome inhibitor, for 2 hours before inducing ER exit. (B) Halo-APP-mNeonGreen RUSH cells were incubated at 20°C to prevent protein export from the Golgi. Processing of Halo-APP-mNeonGreen was monitored for 5 hours after ER export. Single-cell mNeonGreen and Halo-JF646 fluorescence intensities were measured by flow cytometry and expressed as a percentage of the 0 h time point. P^S values indicate the significance of the slope. P^i values indicate the significance of the Y-intercept. ****P≤0.0001; **P≤0.01; ns=not significant.

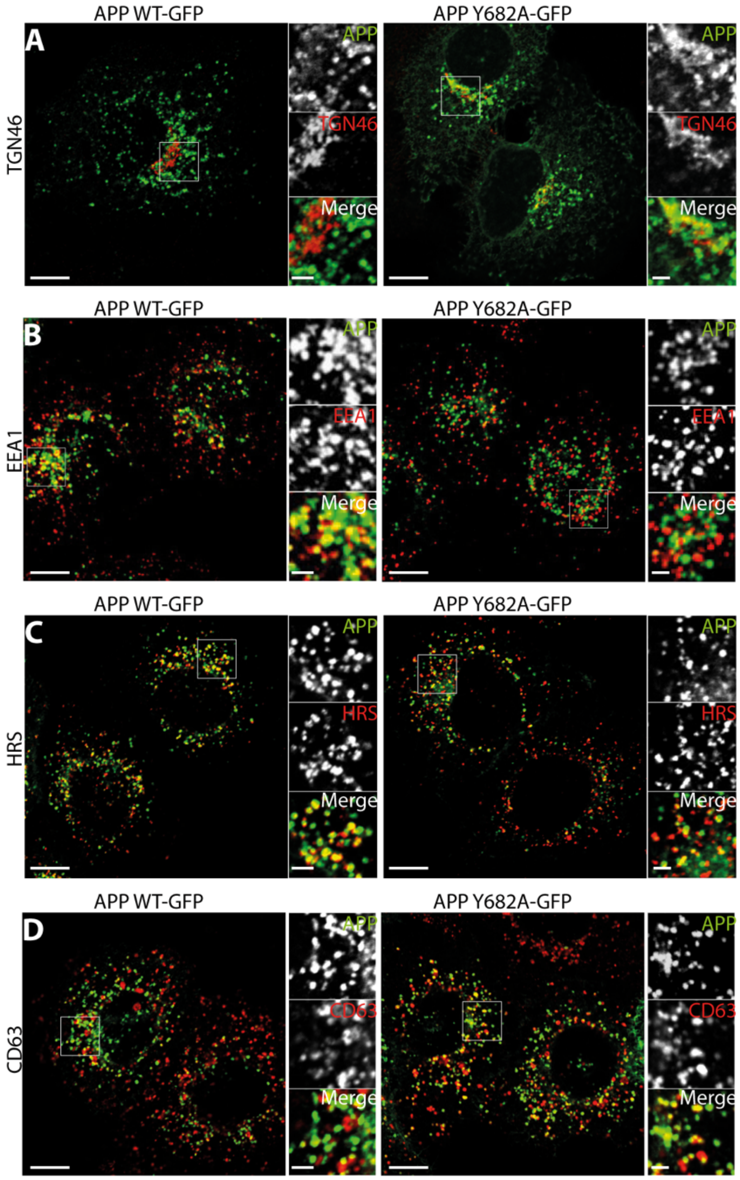

Supplementary Figure 2: KD of AP-1γ1 subunit increases APP association with the TGN46 marker. (A) Confocal microscopy of H4 neuroglioma cells immunolabeled to endogenous APP, γ1 subunit and TGN46. (B) Western blot to confirm efficiency of γ1 subunit KD. H4 cells were submitted to two shots of siRNA knockdown. (C) Confocal microscopy of H4 cells with KD to γ1 subunit, immunolabeled to endogenous APP, γ1 subunit and TGN46. (D) Protein co-localization measured using Fiji software with Pearson's coefficient. Values represent mean ± SEM from at least 11 different cells. *Main panels* scale bar represents 10 μm; *insets* scale bar (2X) represents 2.5 μm. ***p≤0.001. Statistical significance was calculated by a two-tailed Student's t-test.

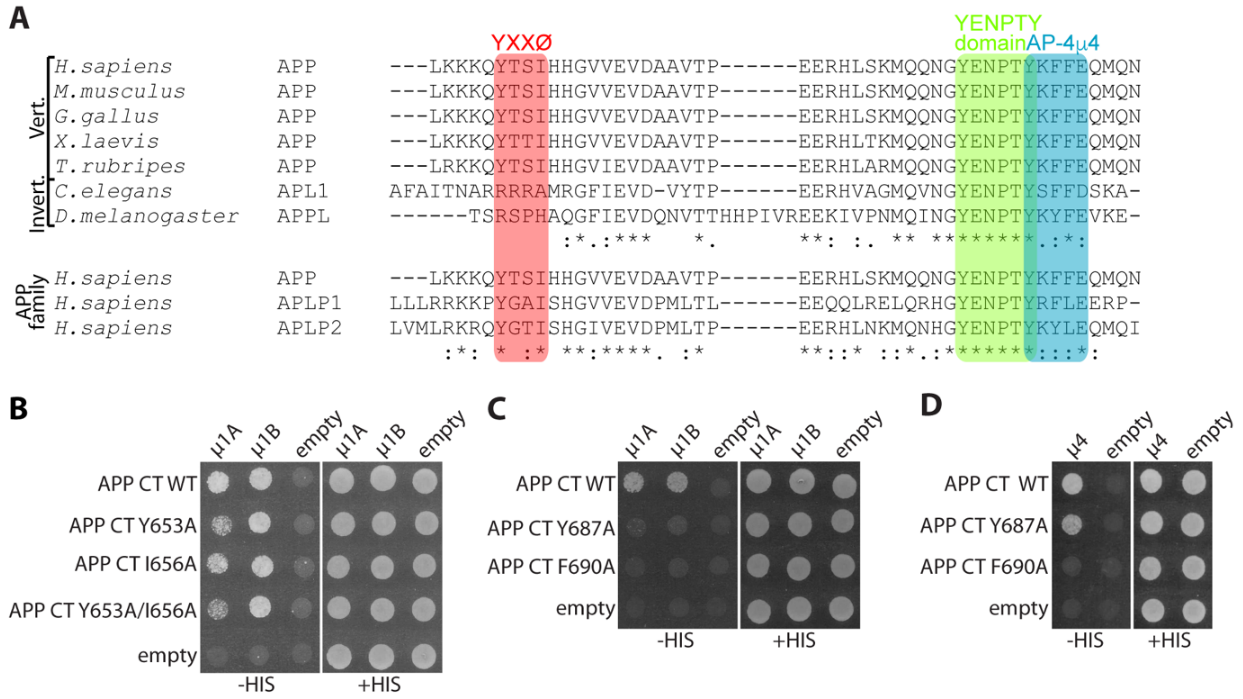

Supplementary Figure 3: APP YKFFE motif mediates its interaction with μ1A, μ1B and μ4. (A) Alignment of APP from vertebrates (Vert.), APP-related genes (APL1 and APPL) from invertebrates (Invert.) and different genes from the APP family in humans (APLP1 and APLP2) showing the conservation and differences present in YXXØ (red), YENPTY (green) and μ4 binding site (blue). (B) Y2H interaction assay between APP with mutations in YXXØ motif (specifically, APP Y653A, APP I656A or APP Y653A/I656A) and μ1A and μ1B. Empty represents the plasmid required for yeast transformation but with no protein expressed. (C) Y2H interaction assay between APP with mutations in YKFFE domain (specifically, APP Y687A and APP F690A), and μ1A and μ1B. (D) Y2H interaction assay between APP with mutations Y687A and F690A, and μ4.

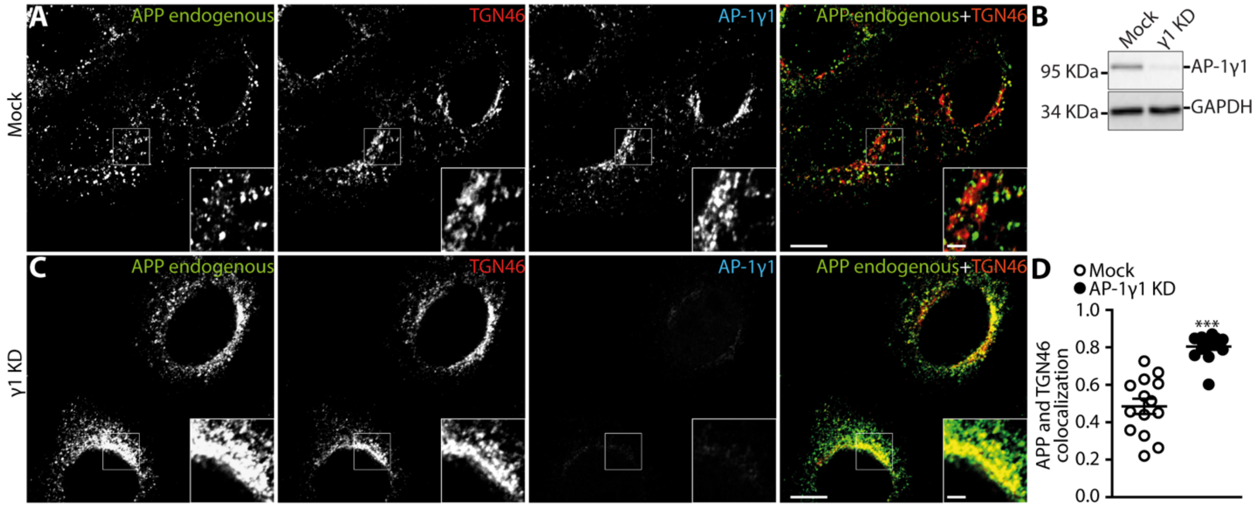

Supplementary Figure 4: APP Y682A is more associated with TGN and late endosomes. APP WT-GFP and APP Y682A-GFP co-transfected in H4 cells and immunolabeled to TGN46 (A), EEA1 (B), HRS (C) and CD63 (D). *Main panels* scale bar represents 10 μm; *insets* scale bar (2X) represents 2.5 μm.

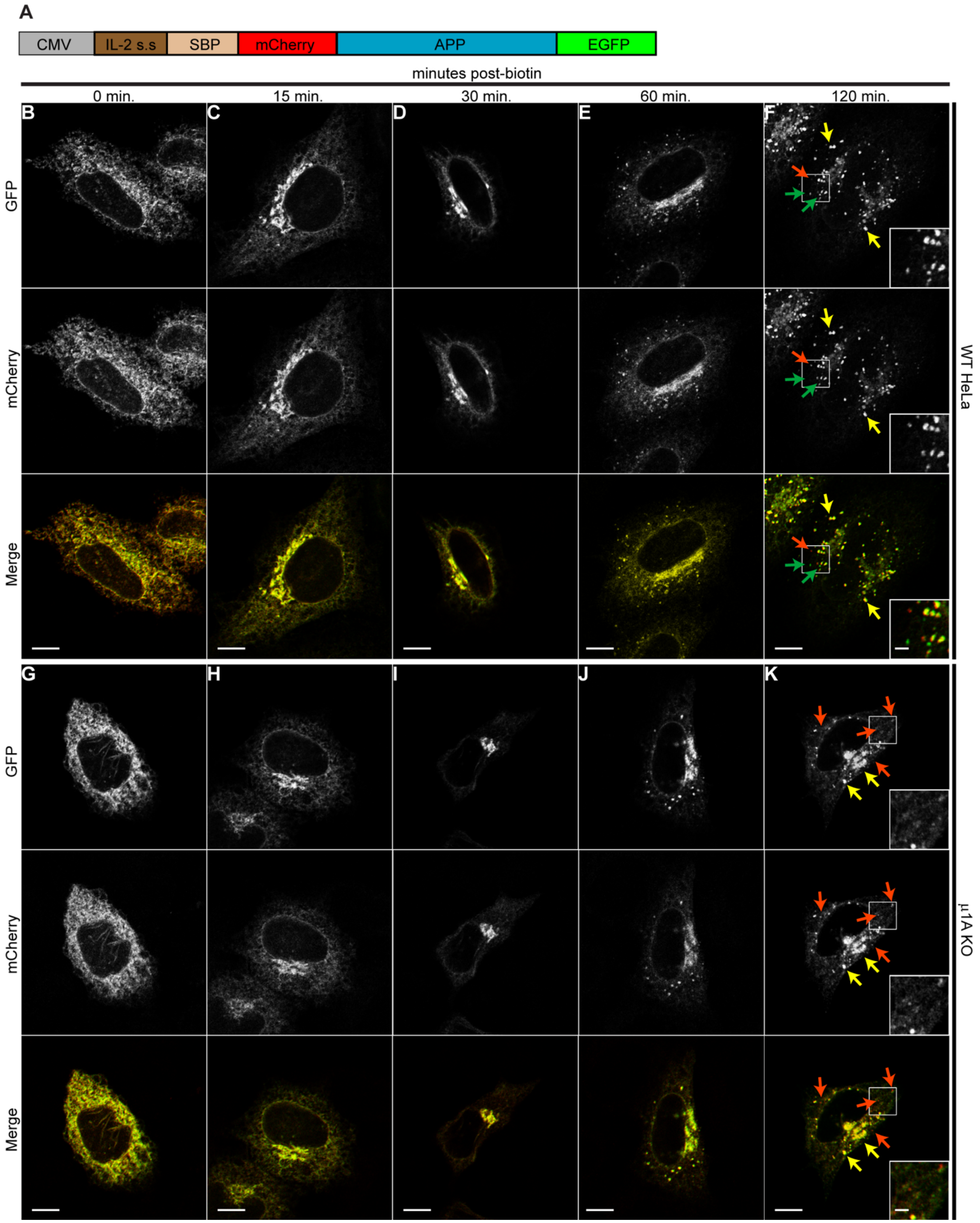

Supplementary Figure 5: APP-RUSH characterization in WT HeLa and µ1A KO cells. (A) Schematic representation of the vector encoding the RUSH mCherry-APP-GFP protein. Streptavidin binding protein

(SBP) binds to Streptavidin-KDEL stably expressed in cells and retains APP in the ER. mCherry is expressed at the N-terminus and GFP expressed at the C-terminus of APP. Treatment with biotin releases APP from Streptavidin-KDEL. WT HeLa (B-F) and µ1A KO cells (G-K) transfected with mCherry-APP-GFP and incubated with soluble biotin for 0, 15, 30, 60 and 120 minutes, as indicated above the panels. Yellow arrows indicate puncta with GFP and mCherry; green arrows indicate puncta enriched with GFP; red arrows indicate puncta enriched with mCherry. *Main panels* scale bar represents 10 µm; *insets* scale bar represents 2.5 µm.
